# Adipocyte-derived shed Syndecan-4 suppresses lipolysis contributing to impaired adipose tissue browning and adaptive thermogenesis

**DOI:** 10.1016/j.molmet.2025.102133

**Published:** 2025-04-01

**Authors:** Jiuyu Zong, Xiaoping Wu, Xiaowen Huang, Lufengzi Yuan, Kai Yuan, Zixuan Zhang, Mengxue Jiang, Zhihui Ping, Lai Yee Cheong, Aimin Xu, Ruby Lai Chong Hoo

**Affiliations:** 1 State Key Laboratory of Pharmaceutical Biotechnology, LKS Faculty of Medicine, The University of Hong Kong, Pokfulam, Hong Kong, China; 2 Department of Pharmacology and Pharmacy, LKS Faculty of Medicine, The University of Hong Kong, Pokfulam, Hong Kong, China; 3 Institute of Digestive Disease and Department of Medicine and Therapeutics, State Key Laboratory of Digestive Disease, Li Ka Shing Institute of Health Sciences, The Chinese University of Hong Kong, Hong Kong, China; 4 Department of Medicine, LKS Faculty of Medicine, The University of Hong Kong, Pokfulam, Hong Kong, China

**Keywords:** Syndecan-4, FGF2, Lipolysis, Energy expenditure, WAT browning

## Abstract

Lipolysis in white adipose tissue (WAT) provides fatty acids as energy substrates for thermogenesis to increase energy expenditure. Syndecan-4 (Sdc4) is a transmembrane proteoglycan bearing heparan sulfate chains. Although single nucleotide polymorphisms (SNPs) of the *Sdc4* gene have been identified linking to metabolic syndromes, its specific function in adipose tissue remains obscure. Here, we show that Sdc4 serves as a regulator of lipid metabolism and adaptive thermogenesis. Sdc4 expression and shedding are elevated in the white adipose tissue (WAT) of diet-induced obese mice. Adipocyte-specific deletion of *Sdc4* promotes lipolysis and WAT browning, thereby raising whole-body energy expenditure to protect against diet-induced obesity. Mechanistically, fibroblast growth factor 2 (FGF2) is a paracrine factor that maintains energy homeostasis. Elevated shed Sdc4 concentrates and delivers FGF2 to fibroblast growth factor receptor 1 (FGFR1) on adipocytes, which in turn suppresses lipolysis by reducing hormone-sensitive lipase (HSL) activity, thus exaggerating adipose tissue dysfunction upon high-fat diet induction. Sdc4-deficient adipocytes show higher lipolytic and thermogenic capacity by enhancing HSL phosphorylation and UCP1 expression. Overall, our study reveals that adipocyte-derived shed Sdc4 is a novel suppressor of lipolysis, contributing to decreased energy expenditure, thus exaggerating obesity. Targeting shed Sdc4 is a potential therapeutic strategy for obesity.

## Highlights

• Diet-induced obesity triggers syndecan 4 (Sdc4) shedding from adipocytes.
• Increased shed Sdc4 impairs adipose tissue browning by suppressing lipolysis.
• Shed Sdc4 activates FGF2/FGFR1 signaling thus reducing HSL activity.
• *Sdc4* deficiency enhances adaptive thermogenesis and alleviates obesity.

## Graphical abstract

**Figure:**
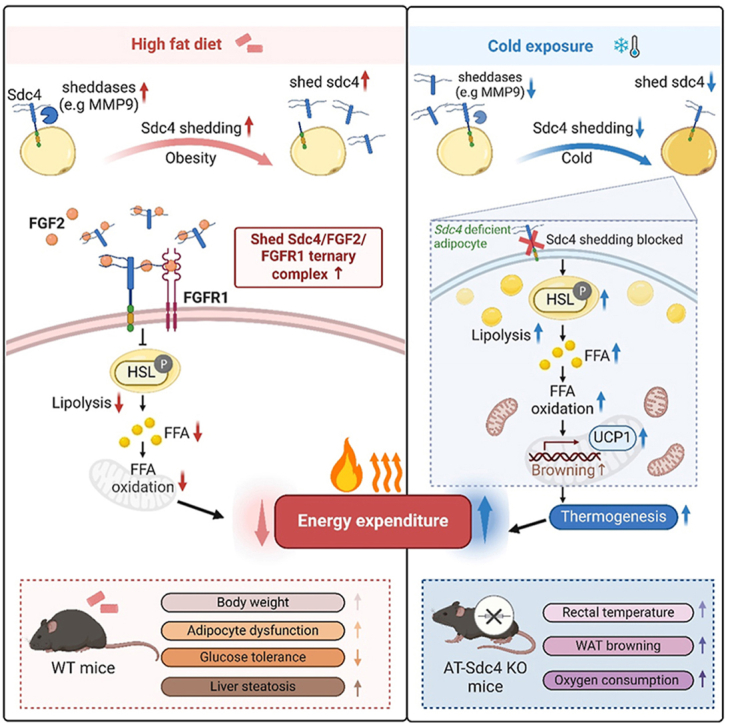

## 1 Introduction

Obesity is mainly attributed to increased calorie intake and/or insufficient energy expenditure, which leads to severe metabolic complications such as diabetes, fatty liver, cardiovascular diseases, and increased cancer risks [[1], [2], [3]]. Bariatric surgery is an effective intervention for treating obesity, but it is only suitable for severely obese individuals and carries high-risk complications [4,5]. FDA-approved anti-obesity medications primarily suppress appetite or fat absorption, while long-term use of these prescription medications may lead to side effects such as vomiting, depression, or muscle loss [[6], [7], [8]]. Enhancing whole-body energy expenditure is always associated with improved systemic metabolism. Hence, identifying drug targets and their signaling pathways involved in enhancing energy expenditure is an urgent need for the discovery of novel interventions to combat obesity.

White adipose tissue (WAT) is an endocrine organ that secretes adipokines and is the major energy reservoir to maintain metabolic homeostasis. The regulation of lipid metabolism in WAT mainly involves lipogenesis, lipolysis, and fatty acid oxidation [9]. Hormone-sensitive lipase (HSL) is a key rate-limiting enzyme controlling lipolysis, and its activity is regulated through the phosphorylation of multiple sites by cAMP-dependent protein kinase A (PKA). Activated HSL then hydrolyzes triacylglycerol (TAG) into diacylglycerol (DAG) and further into monoacylglycerol (MAG) and free fatty acids (FFAs) [10]. In obese individuals, the hypertrophic adipocytes exhibit increased basal lipolysis, and the elevated plasma FFAs contribute to increased ectopic fat deposition, insulin resistance, type 2 diabetes, and related complications [11,12]. However, obesity is associated with blunted catecholamine-stimulated lipolysis and lipid oxidation due to impaired β-adrenoreceptor activation and reduced HSL phosphorylation [13,14]. Contrary to WAT, brown adipose tissue (BAT) acts as a thermogenic organ to promote energy expenditure. Cold exposure stimulates adaptive thermogenesis by activating the sympathetic nervous system (SNS) to release catecholamine, which activates the β_3_-adrenoceptor in BAT to induce uncoupling protein 1 (UCP1) expression and activity for heat generation [15,16]. FFAs released from WAT lipolysis can be oxidized in mitochondria for ATP generation or serve as substrates for UCP1 activation in BAT-mediated thermogenesis [[17], [18], [19], [20]]. Prolonged cold exposure also stimulates WAT browning, which is the recruitment of brown-like adipocytes (called beige adipocytes) expressing UCP1 in subcutaneous WAT (sWAT) to dissipate energy as heat [21]. Due to the relatively small amount of BAT in adult humans, promoting WAT browning is an attractive therapeutic strategy to combat obesity.

Syndecan-4 (Sdc4) is a member of the heparan sulfate proteoglycans (HSPG) family and possesses a core protein with three structural domains: an intracellular domain, a transmembrane domain, and an extracellular domain. Sdc4 modulates cellular functions such as cell adhesion, migration, proliferation, and differentiation [[22], [23], [24]] and is implicated in metabolic diseases. Knockout of *Sdc4* in ApoE^−/−^ mice increases the pro-inflammatory capacity of macrophages and accelerates the development of atherosclerosis [25]. Under normal conditions, the extracellular domain of Sdc4 can be shed and re-expressed for a dynamic balance, while Sdc4 shedding is found elevated under different pathophysiological conditions. Matrix metallopeptidase 9 (Mmp9)-mediated Sdc4 shedding leads to glomerular endothelial glycocalyx damage and increased albumin permeability, exacerbating the development of diabetic kidney disease (DKD) [26]. Increased shed Sdc4 levels in serum are positively correlated with metabolic disorders and NAFLD [27], while reduced serum Sdc4 level was found in individuals with loss of body weight and fat-free mass after bariatric surgery [28]. Single nucleotide polymorphism (SNP) of the human *Sdc4* gene is related to altered fat storage, energy expenditure, insulin sensitivity, and metabolic syndrome [29]. Female *Sdc4* global KO mice fed with a high-fat diet (HFD) display higher levels of circulating triglyceride and impaired insulin sensitivity, while these phenotypes were not observed in male mice [30], indicating potential gender differences in the regulation of metabolism by Sdc4. All these studies indicate a potential function of Sdc4 in the pathogenesis of metabolic disorders. However, its specific role in adipose tissue and how it relates to metabolic disorders remain unclear.

In the present study, the roles of adipocyte-specific Sdc4 and its underlying mechanism in regulating lipolysis in the development of obesity were investigated using adipocyte-specific *Sdc4* knockout mice (AT-Sdc4 KO) and wild-type (WT) littermates. The effect of adipocyte-derived Sdc4 in adaptive thermogenesis in response to cold exposure was also explored.

## 2 Results

### 2.1 HFD feeding upregulates the expression and shedding of Sdc4 in the adipocytes of mice

**Figure 1:**
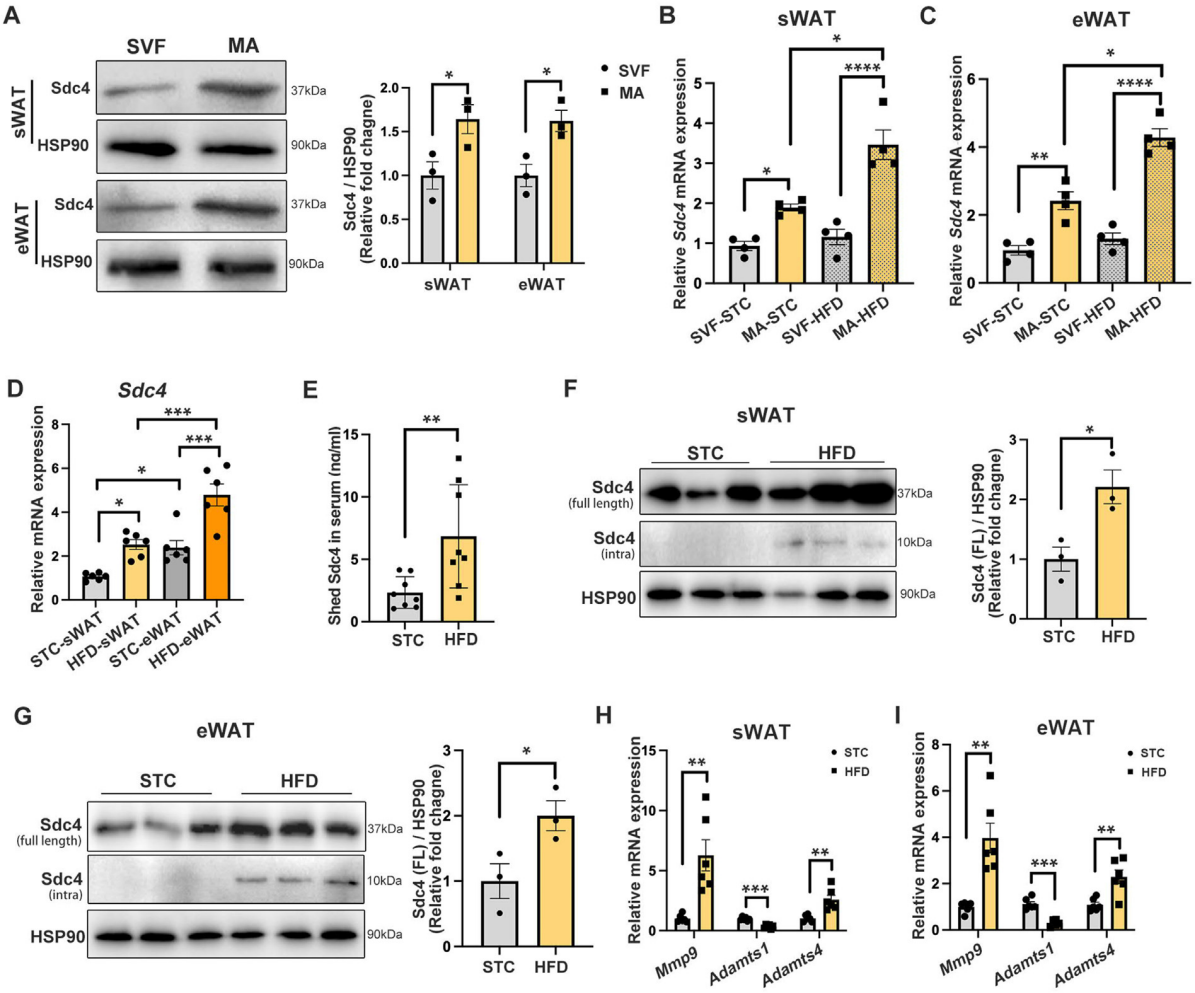
**Sdc4 expression and shedding are increased in adipocytes of obese mice. (A)** Representative immunoblots of protein abundance of Sdc4 and HSP90 in stromal vascular fraction (SVF) and mature adipocytes (MA) in sWAT and eWAT of lean C57BL/6N male mice. The right panel is the quantification of Sdc4 band intensity normalized to HSP90 (n = 3). **(B–I)** Six-week-old C57BL/6N male mice were subjected to standard chow (STC) or high-fat diet (HFD) feeding for 14 weeks. **(B–C)** The relative mRNA abundance of Sdc4 in SVF and MA isolated from mouse (B) sWAT and (C) eWAT (n = 4). **(D)** The relative mRNA abundance of Sdc4 in mouse sWAT and eWAT (n = 6). **(E)** The concentration of shed Sdc4 in mouse serum (n = 8). **(F**–**G)** Representative immunoblots of protein abundance of full-length intact Sdc4, intra Sdc4 (the remaining part after shedding) and HSP90 in mouse (F) sWAT and (G) eWAT. The right panel is the quantification of Sdc4 band intensity normalized to HSP90 (n = 3). **(H–I)** The relative mRNA abundance of Sdc4 sheddase (*Mmp9, Adamts1,* and *Adamts4*) in (H) sWAT and (I) eWAT (n = 6). Data is presented as mean ± SEM. Statistical significance was analyzed by the Mann–Whitney *U* test (A, F, and G), two-way ANOVA (B, C, and D), or unpaired two-tailed Student's *t* test (E, H, and I). ∗*p* < 0.05, ∗∗*p* < 0.01, ∗∗∗*p* < 0.001, ∗∗∗∗*p* < 0.0001.

To investigate the role of Sdc4 in obesity, its expression profile in the WAT of mice under physiological conditions and subjected to diet-induced obesity (DIO) was determined. By isolating mature adipocytes (MA) and stromal vascular fraction (SVF) from subcutanous WAT (sWAT) and epididymal WAT (eWAT), we found that the protein expression of Sdc4 in MA was significantly higher than that in SVF (Figure 1A). Moreover, Sdc4 mRNA expression was further increased in the MA fraction but not SVF in sWAT and eWAT after HFD induction (Figure 1B,C). These findings implicated that the gene expression of Sdc4 is predominantly upregulated in adipocytes under obese conditions. By comparing the Sdc4 mRNA abundance in sWAT and eWAT of mice subjected to standard chow (STC) or HFD feeding, we further found that its mRNA level was significantly higher in eWAT than in sWAT under both conditions (Figure 1D). Regarding Sdc4 protein expression and shedding, shed-Sdc4 concentrations in the serum of HFD-fed mice were significantly increased compared to STC-fed mice (Figure 1E). In parallel, in addition to the induction of full-length (intact) Sdc4 in sWAT and eWAT of HFD-fed mice, the intracellular fragment of Sdc4 (intra-Sdc4), the remaining part of Sdc4 after shedding, was detected in both depots (Figure 1F,G). Concomitantly, the mRNA expression of Sdc4 sheddase, Mmp9 and Adamts4, was significantly induced in both sWAT and eWAT of HFD-fed mice (Figure 1H,I). This evidence further suggests that, along with increased Sdc4 expression, the shedding of Sdc4 is also enhanced in adipose tissue under obese conditions.

By analyzing a single cell RNA sequencing dataset of WAT of mice subjected to STC and HFD feeding [31], we further found that the percentage of *Sdc4*-positive adipocytes significantly increased from from 3.6% to 42.9% in sWAT after HFD feeding (Supplementary Figs. 1A and B), and from 1.2% to 29.9% in eWAT (Supplementary Figs. 1C and D), along with the increased *Sdc4* mRNA levels in adipocytes from both sWAT and eWAT (Supplementary Figs. 1E and F). Given that adipogenesis contributes to fat mass gain during diet-induced obesity, whether Sdc4 is involved in adipocyte differentiation was explored. Along with the induction adipogenic marker PPARγ and FABP4 during 0- to 8-day differentiation, the expression of Sdc4 mRNA and protein were gradually increased (Supplementary Figs. 2A–C). Notably, the gradual increase of intra-Sdc4 protein abundance (Supplementary Fig. 2C) and the significant induction of Mmp9 on day 8 differentiation (Supplementary Fig. 2D) implicated the enhancement of Sdc4 shedding. To determine whether Sdc4 plays a role in adipocyte differentiation, the differentiating profile between SVF-derived WT and Sdc4 KO adipocytes were compared. With no significant Sdc4 mRNA induction during the differentiation of Sdc4 KO adipocytes (Supplementary Fig. 2E), we found that the lipid accumulation and adipogenic marker gene expression were all comparable between WT and Sdc4 KO adipocytes (Supplementary Figs. 2F and G), indicating that Sdc4 is not involved in the adipocyte commitment.

Taken together, these findings indicated that the expression and shedding of Sdc4 in WAT, predominantly from adipocyte fraction, are significantly increased during DIO.

### 2.2 Adipocyte-specific *Sdc4* deficiency protects against diet-induced obesity in mice

Adipose tissue is highly heterogeneous. Since the mRNA and protein levels of Sdc4 were predominantly expressed in mature adipocytes (Figure 1A), the adipocyte-specific *Sdc4* knockout mice (AT-Sdc4 KO) were generated by crossbreeding Sdc4^flox/flox^ mice (WT controls) with adiponectin-promoter-driven Cre transgenic mice to explore the function of Sdc4 in adipose tissue homeostasis (Supplementary Fig. 3A). Both mRNA and protein levels of Sdc4 were significantly decreased in various adipose depots of AT-Sdc4 KO mice, while those in the liver remained unchanged (Supplementary Figs. 3B and C). A small amount of Sdc4 protein expression was still detectable in adipose tissue in AT-Sdc4 KO mice, which could be attributed to its expression in SVFs, the source of preadipocytes and macrophages. No overt abnormalities were observed in AT-Sdc4 KO mice.

**Figure 2:**
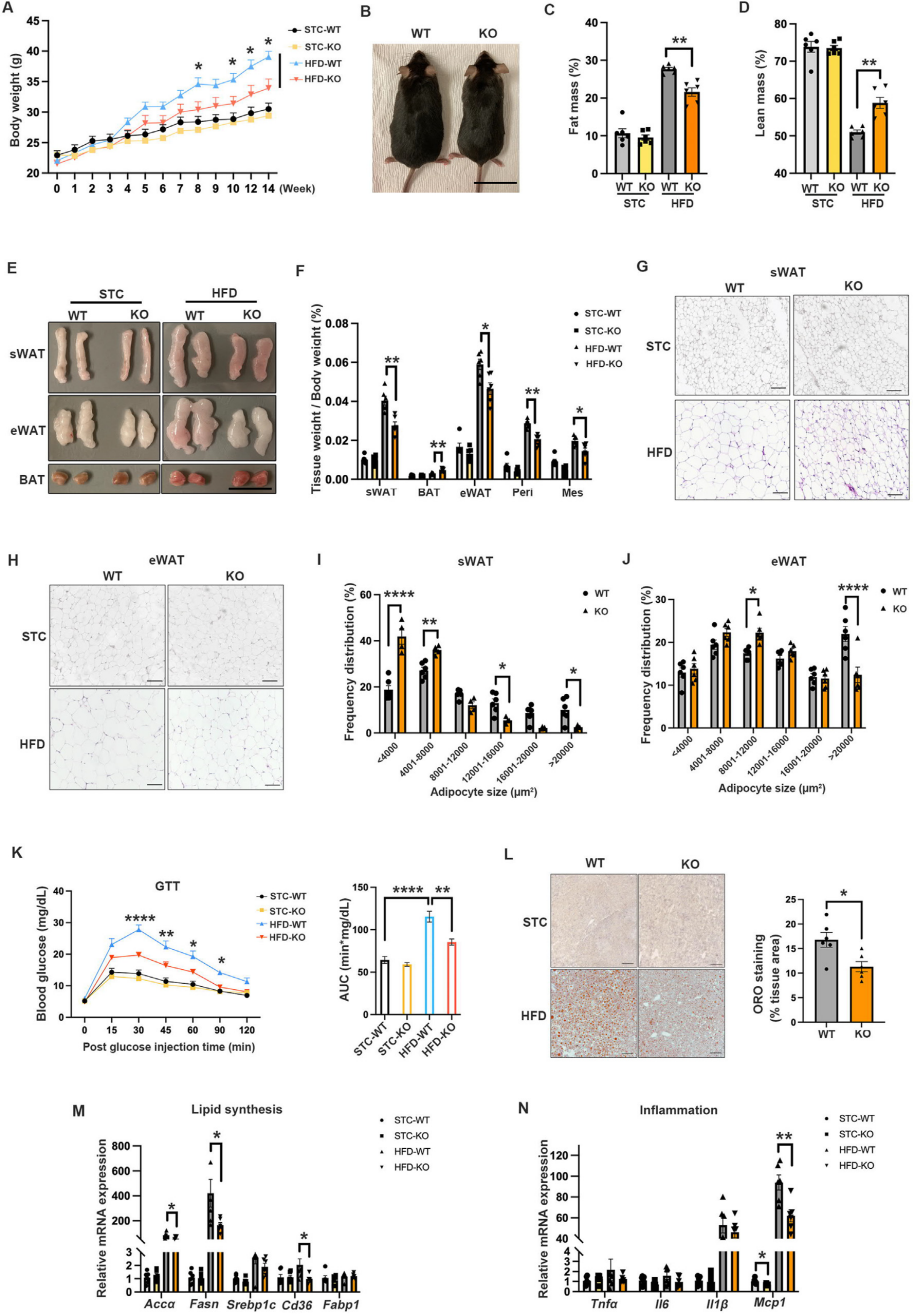
**Adipocyte-specific Sdc4 knockout mice are resistant to diet-induced obesity.** Six-week-old male adipose tissue-specific Sdc4 knockout (AT-Sdc4 KO) mice and relative wild-type (WT) littermates were subjected to HFD or STC feeding for 14 weeks. **(A**–**D)** The (A) body weight, (B) representative images (scale bar = 1 cm), (C) fat mass percentage, and (D) lean mass percentage of AT-Sdc4 KO and WT mice (n = 6). **(E**–**F)** The (E) representative images of mouse sWAT, eWAT, and BAT (scale bar = 1 cm) and (F) relative weights of various fat depots (n = 6). **(G**–**H)** The representative images of H&E staining of mouse (G) sWAT and (H) eWAT (Scale bar = 100 μm). **(I**–**J)** The distribution of adipocytes with different sizes in (I) sWAT and (J) eWAT shown in (G) and (H), respectively (n = 6). **(K)** Glucose tolerance test (left) and calculated areas under the curve (AUC, right) (n = 6). **(L)** The representative images of Oil red O staining of mouse liver. The right panel is the quantification of Oil red O positive area of HFD groups (n = 6). **(M**–**N)** The relative mRNA abundance of genes related to (M) lipid synthesis and (N) inflammation in mouse liver (n = 6). Data is presented as mean ± SEM. Statistical significance was analyzed by two-way ANOVA (A, C, D, F, K, M, and N) or unpaired two-tailed Student's *t* test (I, J, and L). ∗*p* < 0.05, ∗∗*p* < 0.01, ∗∗∗∗*p* < 0.0001. Peri, Perirenal fat; Mes, Mesenteric fat. (For interpretation of the references to color in this figure legend, the reader is referred to the Web version of this article).

Male AT-Sdc4 KO mice and their WT littermates were fed a 45% HFD or STC for 14 weeks. AT-Sdc4 KO mice fed with HFD displayed an attenuated body weight gain and a leaner phenotype compared to WT mice (Figure 2A,B). Body composition analysis revealed a significant reduction in fat mass percentage and an induction in lean mass percentage in HFD-fed AT-Sdc4 KO mice compared to their relative WT littermates (Figure 2C,D). Consistently, HFD-fed AT-Sdc4 KO mice exhibited smaller sizes of sWAT and eWAT (Figure 2E), and the weight of various fat pads was also significantly lower. In contrast, BAT mass was markedly increased (Figure 2F). H&E staining indicated significantly smaller adipocytes in both sWAT and eWAT of HFD-induced AT-Sdc4 KO mice than their relative control mice (Figure 2G,H). The results of adipocyte size quantification further revealed a significantly smaller proportion of large adipocytes in sWAT and eWAT in HFD-induced AT-Sdc4 KO mice compared to their control mice (Figure 2I,J). There were no differences in the above body parameters between AT-Sdc4 KO mice and WT controls under STC feeding (Figure 2A–J). These data demonstrated that adipocyte-*Sdc4* deficiency protects mice against diet-induced obesity.

Obesity is often associated with insulin resistance and hepatic steatosis, exacerbating metabolic dysfunctions [32,33]. The reduced fat accumulation in AT-Sdc4 KO mice prompted us to investigate whether *Sdc4* deficiency in adipocytes could affect the metabolic profile in mice. AT-Sdc4 deficiency significantly alleviated HFD-induced glucose intolerance in mice (Figure 2K), while there were no significant changes in insulin sensitivity among the four mouse groups (Supplementary Fig. 4). Notably, ORO staining of the liver revealed that HFD-fed AT-Sdc4 KO mice displayed a significantly lower hepatic lipid accumulation than HFD-fed WT mice (Figure 2L). The mRNA levels of lipid synthesis- and inflammation-related genes were also significantly decreased in the liver of HFD-fed AT-Sdc4 KO mice, compared to WT controls (Figure 2M,N). Thus, these findings indicated that AT-Sdc4 deficiency attenuates fat mass gain associated with ameliorated obesity-related metabolic disorders in mice.

### 2.3 Adipocyte-specific *Sdc4* deficiency promotes energy expenditure in mice in response to HFD feeding

**Figure 3:**
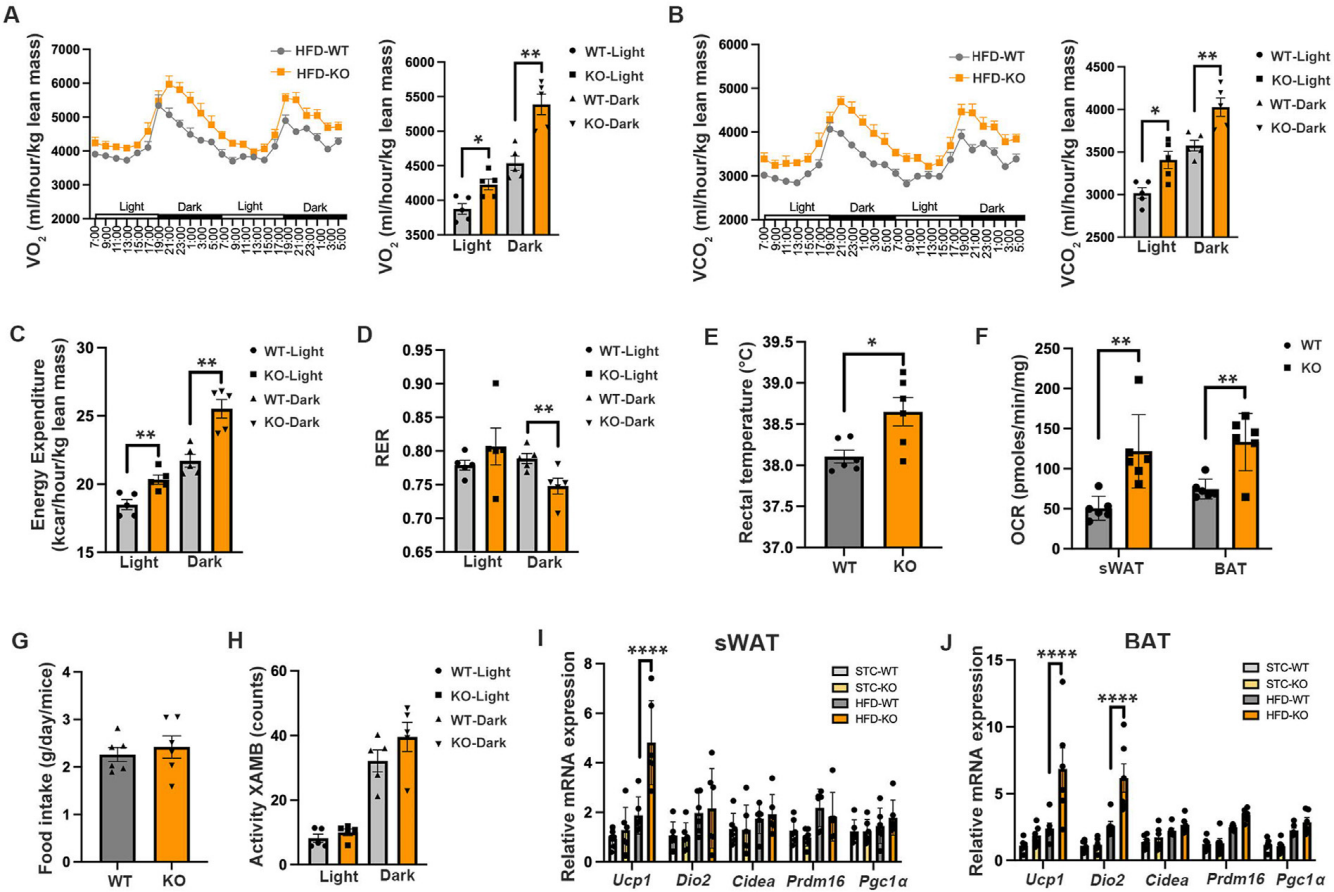
**Adipocyte-Sdc4 deletion elevates energy expenditure in mice in response to diet-induction.** Six-week-old male AT-Sdc4 KO mice and relative WT littermates were subjected to HFD or STC for 14 weeks, followed by metabolic cage assessment at 48 h. **(A**–**B)** The light (day) and dark (night) rate of (A) oxygen consumption (VO_2_) and (B) carbon dioxide production (VCO_2_) in HFD-fed mice. The right panels are the quantification of VO_2_ consumption and VCO_2_ production shown in (A) and (B), respectively (n = 5). **(C**–**D)** The calculated (C) energy expenditure and (D) RER of HFD-fed mice (n = 5). **(E)** Rectal temperature of HFD-fed mice measured at the 48 h endpoint (n = 6). **(F)** The oxygen consumption rate (OCR) of sWAT and BAT from HFD-fed AT-Sdc4 KO and WT mice (n = 6). **(G**–**H)** The (G) food intake and (H) locomotive activity of HFD-fed mice (n = 5). **(I**–**J)** The relative mRNA abundance of thermogenic genes (*Ucp1, Dio2, Cidea, Prdm16,* and *Pgc1α*) in (I) sWAT and (J) BAT of STC- and HFD-fed mice (n = 6). Data is presented as mean ± SEM. Statistical significance was analyzed by unpaired two-tailed Student's *t* test (A–H) or two-way ANOVA (I and J). ∗*p* < 0.05, ∗∗*p* < 0.01, ∗∗∗∗*p* < 0.0001.

Increased energy expenditure is associated with improved glucose and lipid metabolism [34,35]. Thus, the whole-body energy metabolism of mice was assessed. The rate of oxygen consumption (VO_2_) and carbon dioxide production (VCO_2_), as well as whole-body energy expenditure, were significantly increased in HFD-induced AT-Sdc4 KO male mice compared to those of WT controls (Figure 3A–C). Moreover, the RER value of HFD-fed Sdc4 KO was significantly lower than that of WT mice, indicating an increased utilization of fat as a fuel source (Figure 3D). Consistently, HFD-induced AT-Sdc4 KO mice exhibited a significantly higher rectal temperature than WT mice (Figure 3E), along with an increased oxygen consumption rate (OCR) in both sWAT and BAT (Figure 3F), which suggested that adipocyte-Sdc4 deficiency enhanced the rate of mitochondrial respiration. However, there was no difference in the food intake or locomotive activity between AT-Sdc4 KO mice and their WT littermates (Figure 3G,H). Under STC-fed conditions, the rate of VO_2_ and VCO_2_, energy expenditure, rectal temperature, food intake, and locomotive activities were comparable between male AT-Sdc4 KO mice and their WT littermates (Supplementary Fig. 5).

As diet-induced thermogenesis contributes to energy expenditure, the expression of thermogenic markers in mouse WAT and BAT was explored. Under HFD feeding conditions, the Sdc4 deficiency led to increased mRNA expression of the *Ucp1* in sWAT and both *Ucp1* and *Dio2* in BAT (Figure 3I,J). Given that diet-induced thermogenesis is generally less pronounced than the robust response triggered by cold exposure and primarily occurs through BAT [36], we further investigated the UCP1 expression and morphological change of BAT. Under HFD-fed conditions, Sdc4 deficiency enhanced the protein expression of UCP1 in mouse BAT but not sWAT (Supplementary Figs. 6A and B) and reduced lipid accumulation in BAT (Supplementary Fig. 6C). This evidence implicating the enhanced diet-induced thermogenesis in BAT may contribute to the enhanced energy expenditure identified in AT-Sdc4 KO mice.

HFD also induced obesity in female WT and AT-Sdc4 KO mice. However, no significant changes in body weight and body composition, glucose tolerance, and energy expenditure were observed between the WT and AT-Sdc4 KO mice (Supplementary Figs. 7A–I), which may be due to minimal changes of Sdc4 protein expression level in sWAT and perigonadal white adipose tissue (pWAT) of mice subjected to STC and HFD feeding (Supplementary Figs. 7J–K). These data indicate that there is a gender difference in the beneficial effect of AT-Sdc4 deficiency on alleviating obesity-related metabolic syndrome. Male mice were, therefore, used in the subsequent studies. Collectively, these findings suggest that adipocyte-specific Sdc4 deficiency in male mice alleviates diet-induced abnormalities in glucose and lipid metabolism, which may be attributed to enhanced energy expenditure.

### 2.4 Adipocyte-specific *Sdc4* deficiency increases adipocyte lipolysis

**Figure 4:**
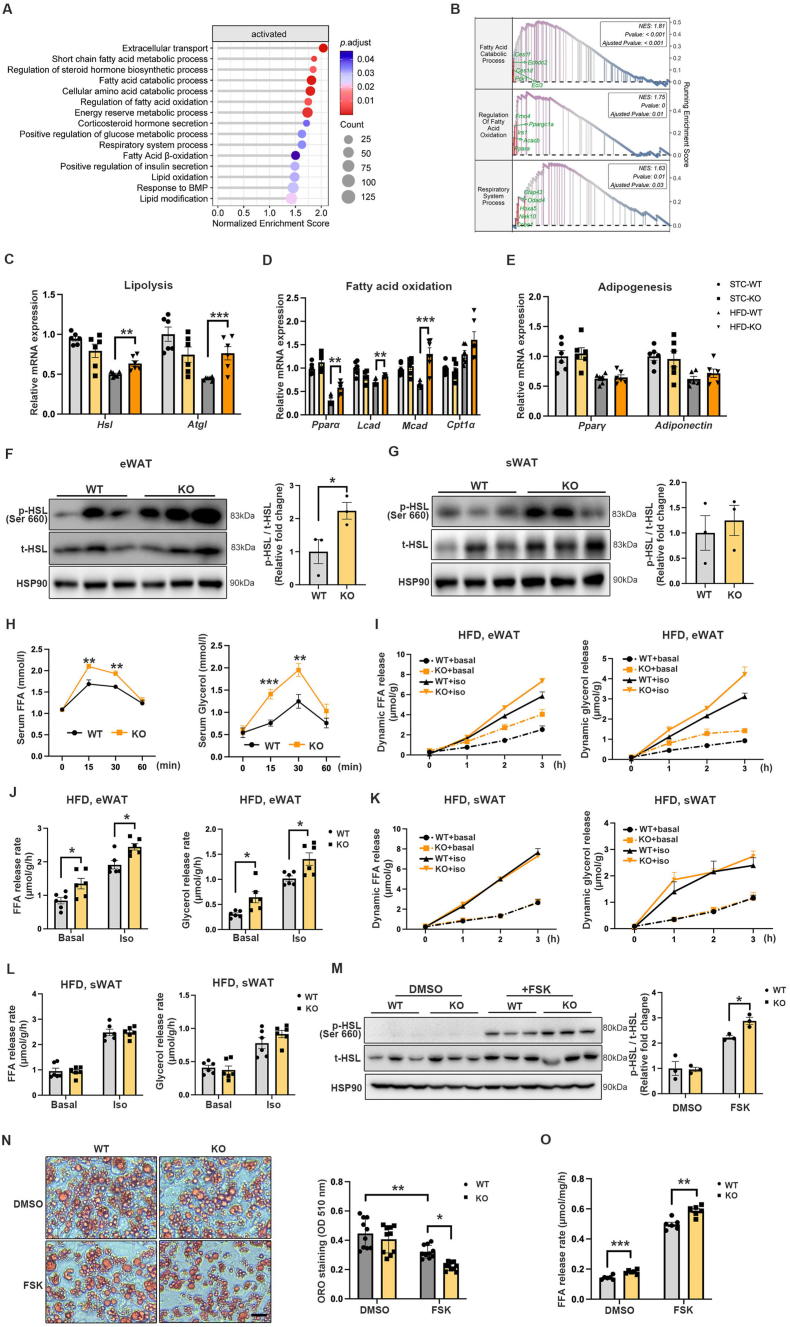
**Sdc4 deficiency in adipocytes promotes lipolysis. (A)** Gene set enrichment analysis (GSEA) of ontology-biological process pathways preferentially activated in the eWAT of HFD-fed AT-Sdc4 KO mice compared to WT mice (n = 4). Terms were ranked based on the normalized enrichment score (NES). **(B)** The representative GSEA-scoring plots of activated GO-BP pathways in (A). The reported NES values and adjusted p values (p. adjust) were calculated with 1,000 permutations in the GSEA software. (**C-E**) The relative mRNA abundance of genes-related to (C) lipolysis, (D) fatty acid oxidation, and (E) adipogenesis in eWAT from AT-Sdc4 KO mice and WT mice subjected to STC or HFD feeding for 14 weeks (n = 6). **(F**–**G)** Representative immunoblots of the protein abundance of p-HSL, t-HSL, and HSP90 in (F) eWAT and (G) sWAT of HFD-fed AT-Sdc4 KO mice and WT littermates subjected to STC or HFD feeding for 14 weeks following fasting for 16 h. The right panel is the quantification of p-HSL band intensity normalized to t-HSL (n = 3). **(H**–**L)** Six-week-old male AT-Sdc4 KO mice and WT mice fed with 14 weeks HFD were injected with isoproterenol (Iso, 10 mg/kg body weight). (H) The serum FFAs and glycerol levels were measured at different time points, as indicated (n = 6). The (I) dynamic and (J) calculated FFA and glycerol releasing profile from eWAT (n = 6). The (K) dynamic and (L) calculated FFA and glycerol releasing profile from sWAT (n = 6). **(M**–**O)** Adipocytes differentiated from SVFs isolated from AT-Sdc4 KO or WT mice were treated Forskolin (FSK, 10 μM) or its vehicle DMSO for 4 h. **(M)** Representative immunoblots of the protein abundance of p-HSL, t-HSL, and HSP90 in adipocytes. The right panel is the quantification of p-HSL band intensity normalized to t-HSL (n = 3). **(N)** The representative images of Oil red O staining of adipocytes (scale bar = 40 μm). The right panel is the quantification of neutral lipid contents (n = 10). **(O)** FFA-releasing rates of adipocytes normalized to protein abundance (n = 6). Data is presented as mean ± SEM. Statistical significance was analyzed by two-way ANOVA (C, D, E, J, L, M, N, and O), unpaired two-tailed student't *t* test (H), or the Mann–Whitney *U* test (F and G). ∗*p* < 0.05, ∗∗*p* < 0.01, ∗∗∗*p* < 0.001. (For interpretation of the references to color in this figure legend, the reader is referred to the Web version of this article).

To investigate the potential mechanism by which Sdc4-deficiency improves obesity-related metabolic syndrome, eWAT was isolated from HFD- or STC-fed AT-Sdc4 KO mice and their WT littermates for RNA-seq. Principal component analysis (PCA) demonstrated a substantial shift in the transcriptome of the HFD-fed WT and AT-Sdc4 KO samples (Supplementary Figs. 8A and B), with 243 up-regulated genes and 482 down-regulated genes differentially expressed between the two groups while the gene expression profiles in STC-fed WT and AT-Sdc4 KO mice were similar, with only five up-regulated genes and two down-regulated genes (Supplementary Fig. 8C). These data suggest that Sdc4 plays a significant role in HFD-induced obesity. Gene Set Enrichment Analysis (GSEA) revealed that those up-regulated genes were most significantly enriched in lipolysis, fatty acid oxidation, and respiratory system processes (Figure 4A,B). The down-regulated genes were enriched in the inflammation-related pathways (Supplementary Figs. 8D and E). RT-qPCR results confirmed that HFD-induced suppression of lipolysis-related (*Hsl and Atgl*) (Figure 4C) and fatty acid oxidation-related genes (*Pparα, Lcad, and Mcad*) (Figure 4D) was significantly alleviated in AT-Sdc4-deficient mice, while *Sdc4* deficiency in adipocytes had no effect on adipogenesis-related gene expression (*Pparγ* and *Adiponectin*) (Figure 4E). Consistently, HFD-induced inflammation-related markers (*Tnfα* and *Mcp1*) were substantially decreased in the eWAT of AT-Sdc4 KO mice (Supplementary Fig. 8F). Short-chain fatty acids (SCFAs) have been associated with improved glucose homeostasis, blood lipid profiles, and enhanced energy expenditure [[37], [38], [39]]. It was noticed that SCFAs metabolism-related genes were also activated in eWAT of AT-Sdc4 KO mice (Figure 4A), which may support the enhanced fatty acid oxidation [40] while the concentrations of the three most common SCFAs (butyrate, acetate and propionate) in the serum were comparable between the WT and AT-Sdc4 KO groups under HFD feeding (Supplementary Fig. 9).

Hormone-sensitive lipase (HSL) is known to mediate lipolysis in mammalian adipocytes [41]. Phosphorylation of HSL in eWAT, but not sWAT, of HFD-fed AT-Sdc4 KO mice was significantly increased compared to that of WT mice (Figure 4F,G). To further investigate whether *Sdc4* deficiency in adipocytes positively regulates lipolysis, 14-week STC- or HFD-fed AT-Sdc4 KO and WT mice were injected with β-adrenergic agonist isoproterenol (ISO, 10 μM) to stimulate lipolysis by activating the protein kinase A (PKA) pathway [42]. Circulating free fatty acid (FFA) and glycerol levels were significantly higher in AT-Sdc4 KO than in their WT control mice within 30 min after ISO injection (Figure 4H), indicating that AT-Sdc4 deficiency promotes lipolysis. To directly measure lipolysis in adipose tissue, eWAT and sWAT from HFD- or STC-fed AT-Sdc4 KO and WT mice were freshly isolated and followed by ISO stimulation for 3 h. FFA and glycerol release rates were significantly increased in eWAT explants of HFD-fed AT-Sdc4 KO mice compared with those of WT controls (Figure 4I,J). FFA release rates were also elevated in the eWAT of STC-AT-Sdc4 KO mice (Supplementary Figs. 10A and B). However, FFA- and glycerol-releasing rates in sWAT were comparable between the WT and AT-Sdc4 KO groups under STC or HFD conditions (Figure 4K, L, and Supplementary Figs. 10C and D). This may be due to higher Sdc4 expression levels in eWAT when compared to sWAT (Figure 1D), and higher androgen receptor (AR) expression [43] and AR-mediated lipolytic activity [44] in visceral fat depots.

In light of the heterogenicity of adipose tissues, SVFs were isolated from WAT of AT-Sdc4 KO and WT control mice and differentiated into white adipocytes *in vitro*, followed by forskolin (FSK, 10 μM) stimulation for 4 h. Immunoblotting results showed that FSK-mediated HSL phosphorylation was significantly upregulated in adipocytes of both groups while the magnitude in *Sdc4* knockout adipocytes was much higher than WT adipocytes (Figure 4M), associating with a decreased number and size of lipid droplets (Figure 4N) and a significantly exacerbated FSK-stimulated FFA release into the medium (Figure 4O). Collectively, these results revealed that Sdc4 deficiency in adipocytes promoted lipolysis, which may explain, at least in part, the reduced fat mass in AT-Sdc4 KO mice.

### 2.5 Adipocyte-specific *Sdc4* deficiency promotes sWAT browning and BAT activation associated with UCP1 upregulation

**Figure 5:**
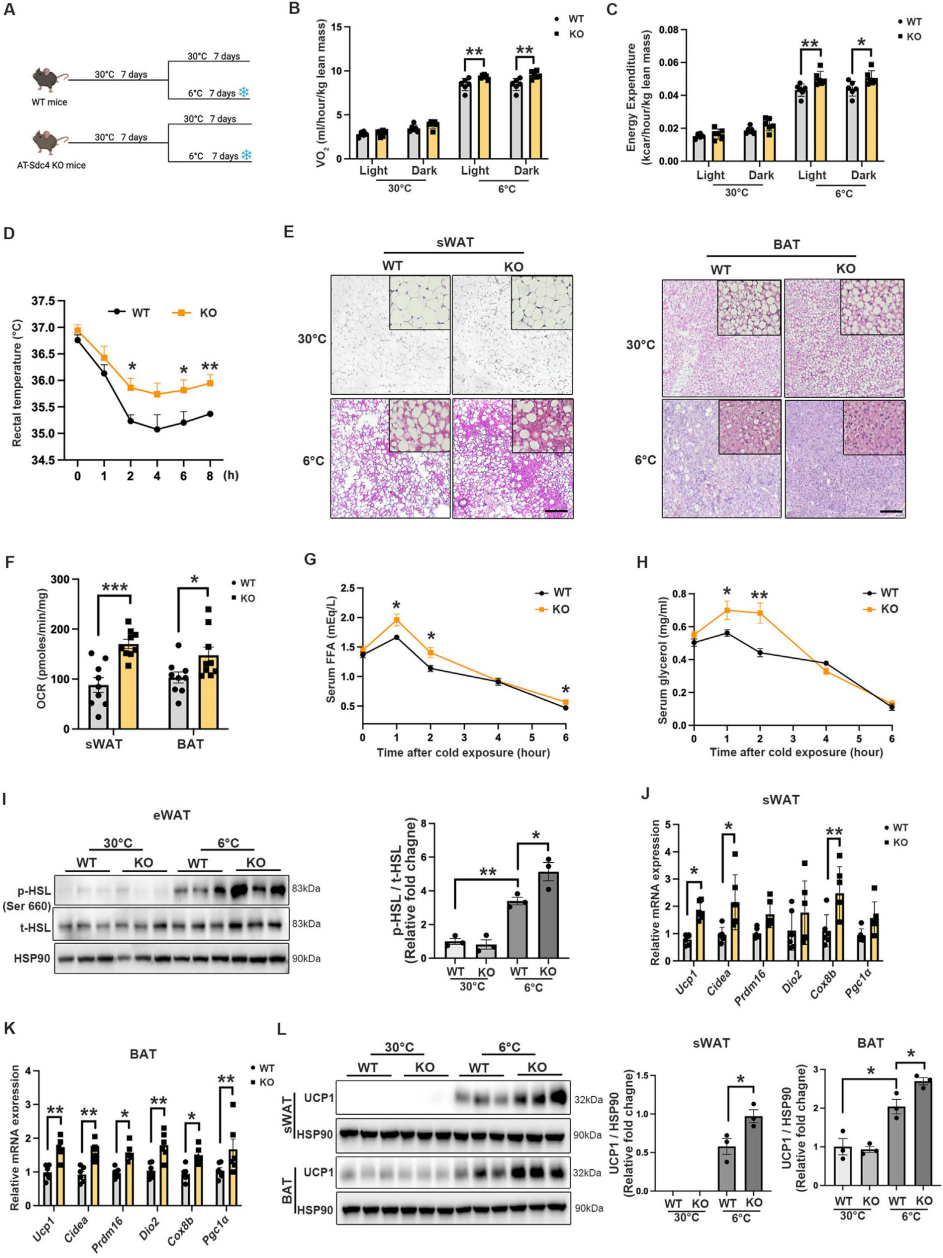
**Sdc4 deficiency in adipocytes promotes sWAT browning and BAT activation.** Eight-week-old male AT-Sdc4 KO mice and their WT littermates were subjected to 30 °C acclimation for 7 days followed by 6 °C cold challenge or 30 °C thermoneutral conditions for 7 days. **(A)** Schematic diagram showing the cold challenge experiments. **(B–C)** The quantification of the (B) rate of O_2_ consumption and (C) energy expenditure of mice in light (day) and dark (night) (n = 6). **(D)** The dynamic rectal temperature of AT-Sdc4 KO mice and WT mice (n = 6) recorded during cold challenge for 8 h. **(E)** The representative images of H&E staining of sWAT (left panel) and BAT (right panel) (scale bar = 100 μm). **(F)** The oxygen consumption rate (OCR) of sWAT and BAT from mice subjected to 6 °C. **(G**–**H)** The serum concentration of (G) FFA and (H) glycerol of mice at indicated time points during cold exposure (n = 6). **(I)** Representative immunoblots of the protein abundance of p-HSL, t-HSL, and HSP90 in mouse eWAT. The right panel is the quantification of p-HSL band intensity normalized to t-HSL (n = 3). **(J**–**K)** The relative mRNA abundance of thermogenic genes in mouse (J) sWAT and (K) BAT (n = 5). **(L)** Representative immunoblots of the protein abundance of UCP1 and HSP90 in mouse sWAT and BAT. The right panels are the quantification of UCP1 band intensity normalized to HSP90 (n = 3). Data is presented as mean ± SEM. Statistical significance was analyzed by two-way ANOVA (B, C, D, G, H, I, and L) or unpaired two-tailed Student's *t* test (F, J and K). ∗*p* < 0.05, ∗∗*p* < 0.01, ∗∗∗*p* < 0.001.

Lipolysis in WAT is an essential process for providing FFA substrate for cold-induced thermogenesis [45]. To determine if AT-Sdc4 deficiency promoted energy expenditure through upregulating WAT browning or BAT activation, STC-fed AT-Sdc4 KO mice and their WT littermates were exposed to 6 °C in a cold chamber for 7 days (Figure 5A), and the metabolic rate in AT-Sdc4 KO and WT mice was assessed. The rate of oxygen consumption (VO_2_) and energy expenditure were significantly higher in AT-Sdc4 KO mice than in WT mice (Figure 5B,C). In addition, AT-Sdc4 KO mice maintained a markedly higher core body temperature than their WT controls throughout the 8 h cold stimulation (Figure 5D), indicating that *Sdc4* deficiency in adipocytes enhanced the thermogenic capacity of mice to dissipate more energy as heat. H&E staining showed that there were more multilocular structures and smaller lipid droplets in sWAT and BAT of AT-Sdc4 KO mice compared with those of WT mice upon cold challenge (Figure 5E). The OCR in isolated sWAT and BAT of AT-Sdc4 KO mice was also significantly increased compared to that of WT mice (Figure 5F), suggesting the enhanced mitochondrial function in Sdc4-deficient adipocytes contributes to sWAT browning and BAT activation. Moreover, the released FFAs and glycerol concentrations in serum were higher in AT-Sdc4 KO mice upon cold stimulation compared to WT littermates and was also accompanied by increased HSL phosphorylation in eWAT (Figure 5G–I). The mRNA expression levels of thermogenic genes were also increased in both sWAT and BAT of AT-Sdc4 KO mice compared with WT controls (Figure 5J,K). Consistently, the UCP1 protein levels were substantially elevated in sWAT and BAT of AT-Sdc4 KO mice than those of their relative WT controls (Figure 5L). These data indicated that the improved cold tolerance and increased energy expenditure in AT-Sdc4 KO mice were attributed to lipolysis-induced UCP1 activation in sWAT browning and BAT activation.

**Figure 6:**
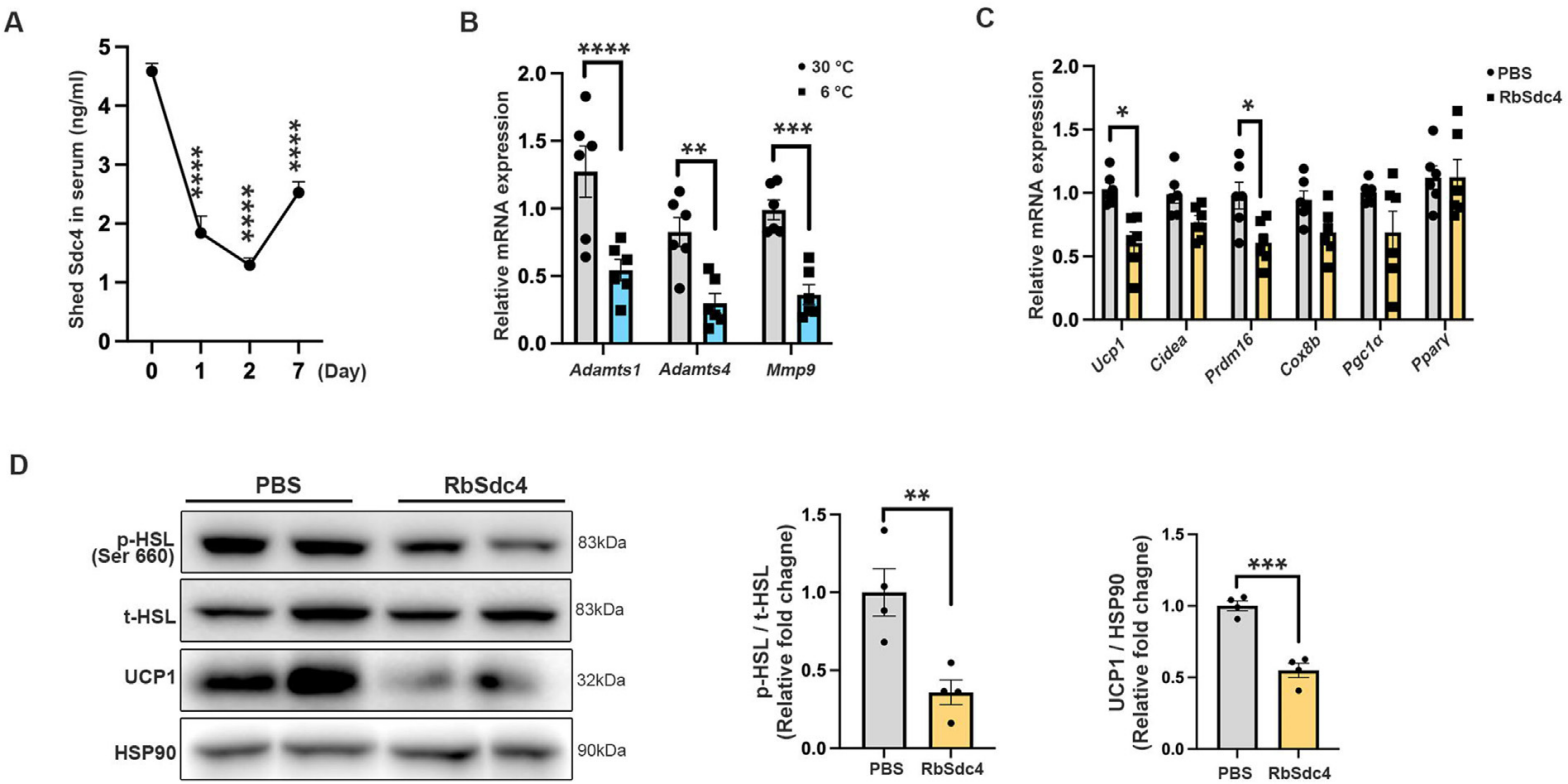
**Sdc4 shedding is inhibited during cold exposure, and recombinant Sdc4 (RbSdc4) inhibits thermogenesis. (A**–**B)** Eight-week-old C57BL/6N male mice were subjected to cold exposure for 7 days. **(A)** The concentration of shed Sdc4 in mouse serum during cold exposure (n = 6). **(B)** The relative mRNA abundance of Sdc4 sheddase in mouse sWAT (n = 6). **(C**–**D)** Beige adipocytes differentiated from SVFs isolated from C57BL/6N mice were treated with either recombinant Sdc4 extracellular domain protein (RbSdc4) or PBS for 24 h. **(C)** The relative mRNA abundance of thermogenic genes (*Ucp1, Cidea, Prdm16,* and *Cox8b*) and adipogenic markers (*Pgc1α* and *Pparγ*) in beige adipocytes (n = 6). **(D)** Representative immunoblots of the protein abundance of p-HSL, t-HSL, UCP1, and HSP90 in beige adipocytes. The right panels are the quantification of p-HSL band intensity normalized to t-HSL and UCP1 band intensity normalized to HSP90 (n = 4). Data is presented as mean ± SEM. Statistical significance was analyzed by one-way ANOVA (A), unpaired two-tailed Student's *t* test (B and C) or the Mann–Whitney *U* test (D). ∗*p* < 0.05, ∗∗*p* < 0.01, ∗∗∗*p* < 0.001, ∗∗∗∗*p* < 0.0001.

We further determined whether Sdc4 shedding was involved in cold-induced thermogenesis. The ELISA results showed that shed Sdc4 concentration was significantly decreased in C57BL/6N mice subjected to cold exposure within 7 days (Figure 6A). By comparing the expression of Sdc4 and its sheddases in the major adipose depots (sWAT, eWAT, and BAT), we found that although the expression of Sdc4 were significantly increased in the sWAT and BAT of cold-exposed mice (Supplementary Figs. 11A–E), the expression of Sdc4 sheddases (*Adamts 1*, *Adamts 4*, and *Mmp9*) in sWAT, the depot with highest Sdc4 mRNA induction, were all reduced (Figure 6B). These data suggest cold induction suppresses Sdc4 shedding from adipocytes. To determine if shed Sdc4 alters thermogenesis, SVF isolated from sWAT of WT mice were differentiated to beige adipocytes and treated with recombinant shed Sdc4 protein (RbSdc4). The mRNA levels of thermogenic genes, as well as HSL phosphorylation and UCP1 protein levels, were markedly decreased in RbSdc4-treated beige adipocytes (Figure 6C,D). Collectively, these findings indicating Sdc4 shedding negatively regulates thermogenesis, which is associated with impaired HSL activity.

### 2.6 Shed Sdc4 inhibits HSL phosphorylation through FGF2-FGFR1 signaling in adipocytes

**Figure 7:**
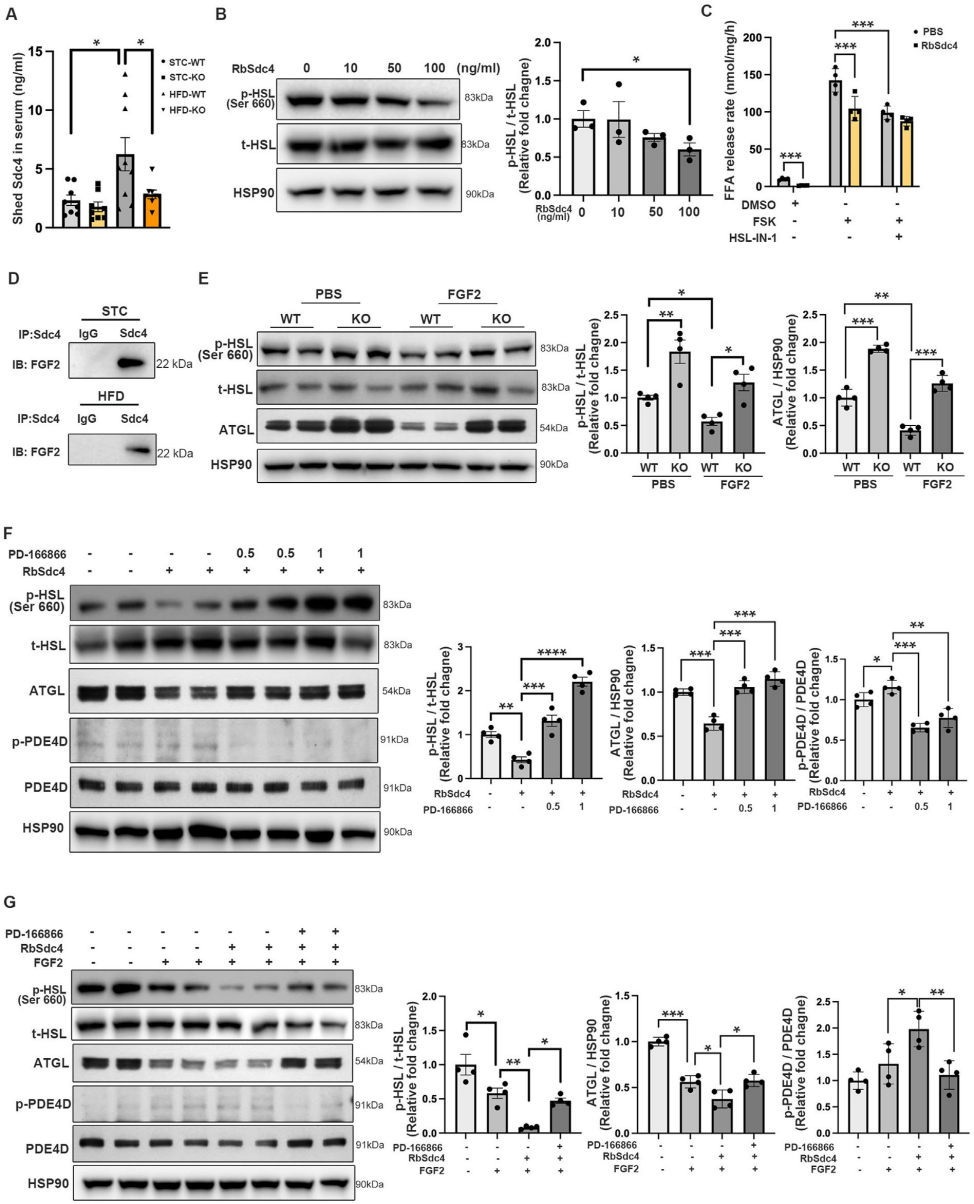
**Shed Sdc4 and FGF2 synergistically inhibit HSL phosphorylation in a FGFR1-dependent manner. (A)** The concentration of shed Sdc4 in serum from AT-Sdc4 KO and WT mice subjected to STC or HFD feeding for 14 weeks (n = 8). **(B)** Representative immunoblots of the protein abundance of p-HSL, t-HSL, and HSP90 in white adipocytes treated with RbSdc4 (0, 10, 50, 100 ng/ml) for 24 h. The right panel is the quantification of p-HSL band intensity normalized to t-HSL (n = 3). **(C)** The FFA release rate of white adipocytes treated with RbSdc4 in the presence or absence of HSL-IL-1 (1 μM) followed by FSK (10 μM) stimulation. The data was normalized by protein abundance (n = 4). **(D)** Representative immunoblot of immunoprecipitation (IP) of Sdc4 and FGF2 in the eWAT of AT-Sdc4 KO mice and WT mice fed with STC or HFD for 14 weeks. **(E)** Representative immunoblots of the protein abundance of p-HSL, t-HSL, ATGL, and HSP90 in Sdc4 KO or WT white adipocytes treated with FGF2 (5 ng/ml) for 24 h. The right panels are the quantification of p-HSL band intensity normalized to t-HSL and the ATGL band intensity normalized to HSP90 (n = 4). **(F)** Representative immunoblots of the protein abundance of p-HSL, t-HSL, ATGL, p-PDE4D, PDE4D, and HSP90 in white adipocytes treated with RbSdc4 (100 ng/ml) in the presence or absence of PD-166866 (0.5 or 1 μM) for 24 h. The right panels are the quantification of p-HSL band intensity normalized to t-HSL, the ATGL band intensity normalized to HSP90, and the p-PDE4D band intensity normalized to PDE4D (n = 4). **(G)** Representative immunoblots of the protein abundance of p-HSL, t-HSL, ATGL, p-PDE4D, PDE4D, and HSP90 in white adipocytes treated with FGF2 (5 ng/ml), RbSdc4 (100 ng/ml), and PD-166866 (1 μM) as indicated for 24 h. The right panels are the quantification of p-HSL band intensity normalized to t-HSL, the ATGL band intensity normalized to HSP90, and the p-PDE4D band intensity normalized to PDE4D (n = 4). Data is presented as mean ± SEM. Statistical significance was analyzed by two-way ANOVA (A, C, E, F, and G) or one-way ANOVA (B). ∗*p* < 0.05, ∗∗*p* < 0.01, ∗∗∗*p* < 0.001, ∗∗∗∗*p* < 0.0001.

Circulating shed Sdc4 was significantly increased in HFD-induced WT mice while that in the HFD-fed AT-Sdc4 KO mice was compromised (Figure 7A). Congruent with enhanced Sdc4 sheddase expression identified in sWAT and eWAT under HFD-fed conditions (Figure 1H,I), this finding suggests that adipocyte is the predominant source of shed Sdc4. To determine whether the shed Sdc4 plays a role in lipolysis, SVFs-differentiated white adipocytes were treated with RbSdc4. We found that RbSdc4 suppressed HSL phosphorylation in a dose-dependent manner (Figure 7B). In parallel, RbSdc4 inhibited FFA release from adipocytes under both basal and forskolin (FSK)-stimulated conditions, which was diminished upon HSL inhibition by its specific inhibitor HSL–IN–1 (Figure 7C). This is in line with the increased lipolysis observed in HFD-fed AT-Sdc4 KO mice (Figure 4).

It has been shown that shed Sdc4 can act as a co-receptor to interact with growth factors and modulate different cellular processes, similar to its extracellular domain of intact Sdc4 [46,47]. Fibroblast growth factor 2 (FGF2) functions in either autocrine or paracrine fashion through binding to fibroblast growth factor receptor 1 (FGFR1) with the highest affinity [48,49]. The activation of FGFR1 signaling suppresses adipose lipolysis via the cAMP/PKA axis, which enhances phosphodiesterase-4 (PDE4) phosphorylation to inhibit HSL activation [50]. The extracellular domain of Sdc4 or shed Sdc4 was shown to bind and deliver FGF2 to its receptor for FGF2 internalization [[51], [52], [53]]. Thus, we investigated if the shed Sdc4 suppresses lipolysis through modulating the FGF2/FGFR1 axis. The results of co-immunoprecipitation (co-IP) assays of adipose tissue lysate indicated that Sdc4 had a strong interaction with endogenous FGF2 under STC conditions, while the interaction was slightly decreased when mice were fed with HFD, which might be due to the increased shedding of Sdc4 upon HFD feeding (Figure 7D). Moreover, FGF2-mediated suppression of HSL phosphorylation and ATGL expression were significantly attenuated in Sdc4 deficient-adipocytes (Figure 7E). These findings implied that FGF2 was a ligand for Sdc4 to regulate lipolysis in adipocytes.

Adipocytes were treated with RbSdc4 and FGFR1-specific inhibitor (PD-166866) to determine whether shed Sdc4-reduced lipolysis in adipocytes was through activating FGFR1. As expected, RbSdc4-stimulated upregulation of PDE4D phosphorylation at PKA site and reduction of HSL phosphorylation and the subsequent suppression on ATGL expression were abolished upon FGFR1 inhibition (Figure 7F). In addition, the co-treatment with RbSdc4 and FGF2 synergistically and significantly activated PDE4D phosphorylation while inhibiting HSL phosphorylation and ATGL expression when compared to FGF2 treatment alone, which was also abolished upon FGFR1 inhibition (Figure 7G). These data not only reinforced the shed Sdc4/FGF2-reduced HSL phosphorylation under obese conditions, but also confirmed the action is mediated through the FGFR1 receptor.

## 3 Discussion

Sdc4 is a transmembrane proteoglycan ubiquitously expressed in various cell types [22], and its genetic variants are strongly associated with intra-abdominal fat, insulin sensitivity index, and triglyceride (TG) levels [30,54,55]. However, the specific function and underlying mechanism of adipocyte-derived Sdc4 in obesity remain elusive. In this study, we identified that adipocyte-derived shed Sdc4 is a novel suppressor of lipolysis leading to attenuated FFA-oxidation, reduced adaptive thermogenesis, and exaggerated adipose tissue dysfunction, thus aggravating obesity. Sdc4 expression and shedding are significantly elevated in the WAT of obese mice. Genetic ablation of Sdc4, specifically in adipocytes, promotes whole-body energy expenditure in mice, associated with enhanced lipolytic capacity and WAT browning, which lowers chronic inflammation and alleviates diet-induced obesity and its related metabolic syndrome. Mechanistically, adipocyte-derived shed Sdc4 promotes FGF2/FGFR1 signaling transduction to inhibit HSL phosphorylation, leading to impaired lipolysis with reduced FFA release from adipocytes. Accumulated lipid provokes adipose tissue dysfunction, and fewer FFAs can be used as the substrate for thermogenesis, thereby exaggerating obesity [11,56]. Hence, these findings highlight the therapeutic potential of targeting shed Sdc4 to alleviate obesity through improving lipid metabolism.

Sdc4 expression and shedding maintain a dynamic balance under physiological conditions, which can be disrupted by pathological stimuli [22]. Elevated Sdc4 shedding has emerged as a risk factor for metabolic disorders. Patients with resistant arterial hypertension and type 2 diabetes present higher serum Sdc4 levels [57]. Circulating Sdc4 levels are positively associated with diabetic kidney disease and non-alcoholic fatty liver disease (NAFLD) [26,27,58]. Increased Sdc4 shedding is observed in patients with atrial fibrillation, which is correlated with atrial oxidative and inflammatory response [59]. Sdc4 shedding also contributes to the progression of cardiac fibrosis by promoting thrombin-cleaved osteopontin-mediated collagen production [60]. The present study is the first time to disclose that shed Sdc4 extracellular domain was considerably increased in the serum of HFD-induced obese mice, while this induction was significantly attenuated in adipocyte-Sdc4 deficient mice associating with reduced body weight gain and alleviated metabolic syndrome, including glucose intolerance and steatohepatitis indicating adipocyte is the primary cellular source of shed Sdc4 which plays a novel role in the pathogenesis of obesity and its related metabolic disorders. Recently, reduced serum Sdc4 level was shown to be associated with loss of body weight and fat-free mass after bariatric surgery [28]. Therefore, circulating shed Sdc4 may serve as a potential biomarker of obesity and its related complications.

Matrix metallopeptidase 9 (Mmp9), one of the critical sheddases of Sdc4, is significantly increased in the adipose tissue of HFD-induced mice and is consistent with previous findings that adipose tissue *Mmp9* mRNA levels are positively correlated with nondiabetic subjects with a higher BMI [61,62]. A strong positive correlation between Mmp9 and shed Sdc4 has been identified in the synovial fluid of osteoarthritis patients, while inhibition or knockdown of Mmp9 reduces Sdc4 shedding [63,64]. Thus, it is highly possible that the elevated Mmp9 levels predominantly mediate the increased Sdc4 shedding observed in adipose tissue under obese conditions. With the fact that increased Sdc4 and Mmp9 have been shown to contribute to obesity-related disorders such as resistant hypertension and atrial fibrillation [57,59,65,66], the detrimental effects of Mmp9 may be at least partially mediated through its induction on Sdc4 shedding. Further investigations are warranted to explore the potential impact of Mmp9 inhibition in mitigating the pathogenic effects of shed Sdc4. On the contrary, the mRNA levels of *Mmp9* and other Sdc4 sheddases, including Adamts1 and Adamts4, were decreased in response to cold exposure, along with the reduced shed Sdc4 levels in serum. These observations suggest that Mmp9 and other sheddases may play a significant role in regulating the pathophysiological effects of Sdc4, highlighting the need for further research to elucidate their contributions to the overall regulation of Sdc4 shedding and its impact on energy metabolism and systemic homeostasis.

Improved metabolic phenotypes including lower body weight and reduced adipose tissue gain and higher energy expenditure in diet-induced AT-Sdc4 KO mice were associated with the markedly increased expression of lipid metabolism-related genes in WAT as revealed by RNA-seq analysis, suggesting a critical role of Sdc4 in lipid metabolism. Consistently, AT-Sdc4 KO mice exhibit elevated HSL-mediated lipolysis and FFA oxidation while treatment with recombinant Sdc4 (RbSdc4) impairs lipolysis in adipocytes by decreasing HSL activity and FFA release indicating that the adipocyte-derived shed Sdc4 of HFD-fed mice acts in an autocrine manner to disrupt lipid metabolism, thereby aggravating the development of obesity. Hepatic shed Sdc4 is an exercise-responsive hepatokine that also works in autocrine manner to reduce lipid storage in hepatocytes [67], which supports our findings on the autocrine effect of shed Sdc4 to suppress lipolysis in adipocytes and reinforces its role in regulating lipid metabolism in various organs.

HSL-catalyzed lipolysis is essential for thermogenesis as the released FFA is the major energy substrates and inducer for UCP1 activation [[68], [69], [70]]. More multilocular lipid droplets and higher oxygen consumption rate were observed in sWAT and BAT of AT-Sdc4 KO mice along with increased HSL phosphorylation in eWAT and a higher rectal temperature in response to cold stimulation. In addition, cold exposure reduced sheddase expression and Sdc4 shedding in adipocytes associating with increased lipolysis, whereas RbSdc4 treated-beige adipocytes exhibited a significant decreased UCP1 expression. These data collectively implicated that targeting shed Sdc4 or suppression of Sdc4 shedding could be a potential intervention for obesity through enhancing lipolysis and the subsequent thermogenic function.

Furthermore, the findings also suggest that shedding of Sdc4 is tightly controlled to mediate adaptive change in response to diet-induction and cold stimulation to maintain metabolic homeostasis. In healthy stage, diet-induced shed Sdc4 suppresses lipolysis to prevent ectopic lipid accumulation. Cold induction suppresses Sdc4 shedding therefore allow lipolysis to provide FFAs as substrates for thermogenesis. However, under pathological conditions such as obesity, chronic elevation of shed Sdc4 leads to the exaggerated inhibition of lipolysis not only impairs thermogenesis but also induces adipose tissue hypertrophy and eventually leads to insulin resistance and metabolic diseases. Regarding the glucose metabolism, AT-Sdc4 KO mice exhibited improvement in the glucose tolerance test but not the insulin tolerance test, which implied that adipose tissue Sdc4 deficiency may improve glucose-induced insulin production. Under T2D conditions, it has been reported that pancreatic FGF2 expression [71], islet FGFR1 expression [71], and circulating Sdc4 levels [26,72] are increased. Treatment of FGF2 induces the dedifferentiation of pancreatic beta cells, reducing the insulin production and secretion capacities [71]. Thus, it is possible that, under obese conditions, adipose tissue released shed Sdc4 also targets the pancreas and reduces insulin secretion by enhancing the FGF2-mediated beta cell dedifferentiation. Further studies are warranted to explore the potential regulation of shed-Sdc4 in beta cell function.

Adipocyte-derived shed Sdc4 suppresses lipolysis by interacting and concentrating FGF2 which then acts on the FGFR1 on the adipocytes in an autocrine manner to inhibit HSL phosphorylation. Shed syndecans are known to be more effective than the cell surface-bound intact syndecans in binding with FGF2 [47,73], and the heparin sulfate (HS) chains in Sdc4 extracellular domain have the strongest binding propensity and high affinity for FGF2 [74,75]. Sdc4 can act as a co-receptor to bind and concentrate FGF2 to prevent its diffusion, thereby strengthening the interaction between FGF2 and its receptor to regulate downstream signals [74,76]. FGF2 has biphasic effects on adipogenesis depending on its concentration [77]. The secretion of FGF2 is increased in HFD-induced obese mice [48] while its global knockout mice are resistant to HFD-induced adiposity and steatosis by increasing UCP1-activated thermogenesis [48], which requires the involvement of heparin. These data are similar to what we observed in adipocyte Sdc4-deficient mice. FGF2 was strongly bound to Sdc4 in WT adipocytes while FGF2-mediated inhibition of HSL phosphorylation was attenuated in Sdc4-deficient adipocytes, suggesting that FGF2 regulates HSL activity via Sdc4. FGFR1 is the most common receptor for FGF2 in various biological processes [48,49], and the activation of FGFR1 inhibits lipolysis by increasing PDE4 phosphorylation, thus reducing HSL phosphorylation in diet-induced obese mice [50]. In this study, shed Sdc4 exacerbated FGF2-mediated inhibition of HSL phosphorylation, but this effect was partly eliminated when adipocytes were treated with FGFR1 inhibitor, suggesting FGFR1 is involved in shed Sdc4/FGF2-mediated suppression of lipolysis. Furthermore, treatment with RbSdc4 suppressed HSL phosphorylation and UCP1 expression in beige adipocytes. Previous studies showed that FGF2-inhibited UCP1 expression was potentially associated with FGFR1 activation [48]. Thus, it is possible that the negative regulatory effect of shed Sdc4 on UCP1 expression may also via FGF2/FGFR1 and further investigations are warranted. Although the presence of a ternary complex of shed-Sdc4, FGF2, and FGFR1 warrants further investigations using approaches such as cryo-electron microscopy, our study is the first to elucidate that the adipocyte-derived shed Sdc4 facilitates the FGF2/FGFR1 signaling transduction. Subsequently, the shed-Sdc4-induced FGF2/FGFR1 activation inhibits adipocyte lipolysis by suppressing HSL phosphorylation, which uncovered a specific mechanism of shed Sdc4 in the development of obesity.

In our study, only male AT-Sdc4 KO mice fed with HFD (45% kcal fat) showed decreased body weight gain, smaller adipocyte size, and higher energy expenditure compared to their relative WT controls, whereas these phenotypes were not pronounced in female AT-Sdc4 KO mice. This may be due to the fact that estrogen inhibits lipolysis by increasing the number of antilipolytic alpha2A-adrenergic receptors in adipocytes [78]. Therefore, highly released estrogen in female mice may counteract the increased lipolytic capacity caused by Sdc4 deficiency in adipocytes, resulting in no significant improvement in lipid metabolism. In addition, some of our findings are contradictory to those in De Luca, M. et al. study in which the fat mass percentage and hepatic triglyceride content were significantly increased in Sdc4 global KO female mice fed with HFD (60% kcal fat) and was not observed in male global KO mice [30]. The potential reasons for the discrepancies are: 1) De Luca, M. et al. study used Sdc4 global KO mice throughout the study [30] while we used adipocyte-Sdc4 KO mice. Since Sdc4 is ubiquitously expressed in various types of cells and can modulate various biological processes [24], global deletion of Sdc4 may alter cellular and systemic metabolism; 2) The basal metabolism of different mouse strains is diverse. They used global Sdc4 KO mice with a C57BL/6J background, which carries the nicotinamide nucleotide transhydrogenase (Nnt) mutation with a lower metabolic rate [79,80]. Our AT-Sdc4 KO mice are in C57BL/6N background, which is more commonly used for metabolic studies; 3) The diet we used to induce obesity contains 45% kcal fat, whereas other studies used 60% HFD or 10% low-fat diet (LFD) [29,30]. Diets with different fat contents may trigger a discrepant response to metabolism due to different hormonal releases [81,82]. Here, the use of adipocyte-Sdc4-KO mice in the present study provides a clear picture of the regulatory role of adipocyte-derived Sdc4 in obesity.

**Figure 8:**
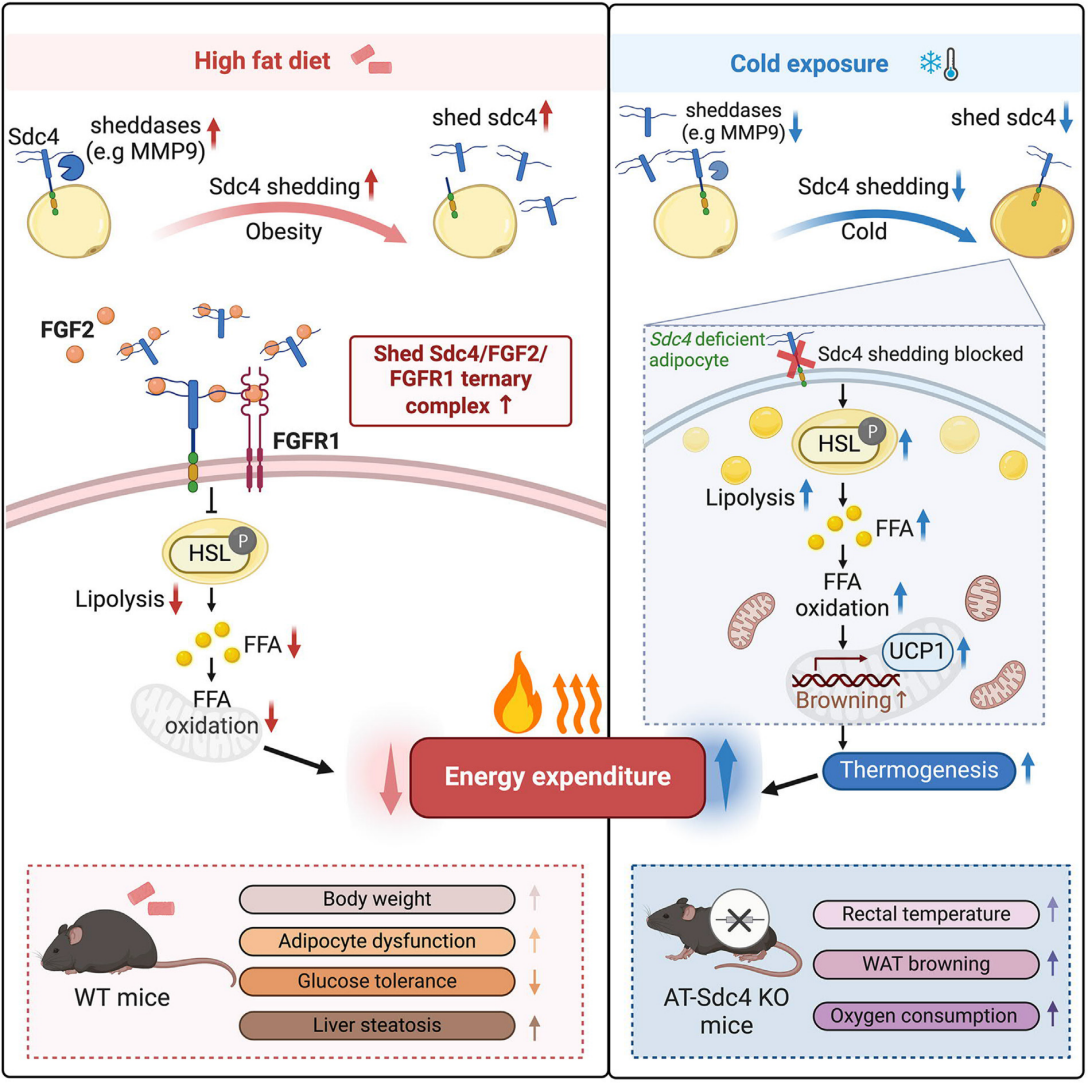
**Proposed summary.** Adipocyte Sdc4 shedding increased in mice subjected to diet-induced obesity. Shed Sdc4 binds and concentrates FGF2, thus activating FGFR1 to suppress HSL phosphorylation. Impaired lipolysis reduces FFA release for FFA oxidation and, therefore, reduces energy expenditure. Accumulated lipid in adipocytes leads to adipose tissue dysfunction. Both of these exaggerate obesity and its related metabolic disorders. In AT-Sdc4 KO mice subjected to cold exposure, lack of Sdc4 shedding waives the suppressing effect on HSL-mediated lipolysis. FFA can, therefore, be provided as an energy substrate for thermogenesis.

In conclusion, evidence from genetic ablation and exogenous treatment in *in vitro* models ofadipocytes demonstrate, for the first time, that shed Sdc4 from adipocytes during obesity acts as a novel suppressor of lipolysis through the FGF2/FGFR1 axis. Impaired lipolysis reduces FFA release, on one hand leading to adipocyte hypertrophy and, on the other hand, leading to reduced substrate for fatty acid oxidation and thermogenesis, attenuating whole-body energy expenditure, which eventually exaggerates obesity and its related metabolic disorders (Figure 8). Given the fact that deletion of Sdc4 in adipocytes abolishes Sdc4 shedding thus attenuating diet-induced body fat gain and other metabolic syndrome, targeting shed Sdc4 is a potential therapeutic strategy for obesity.

## 4 Method

### 4.1 Sex as a biological variable

Sex was consistently considered as a variable. In animal studies, only male adipocyte-specific Sdc4 knockout (AT-Sdc4 KO) mice exhibited improved metabolic phenotypes rather than female mice. This study mainly used male animals.

### 4.2 Animals

Breeding pairs of homozygous Sdc4 floxed mice (Sdc4^f/f^) and adiponectin-Cre mice were purchased by Cyagen (CA, USA), and both were backcrossed with C57BL/6N mice for at least ten generations. Adipocyte-specific Sdc4 knockout (AT-Sdc4 KO) mice and their wild-type (WT) littermates were generated by crossing Sdc4^f/f^ mice with adiponectin-Cre mice, and age-matched male mice were used in all the studies. All animals were housed in the conventional experimental holding area (CA-FMB) at the University of Hong Kong, following a 12-hr light/12-hr dark cycle, and maintained at a humidity level of 60–70%. They were provided with free access to food and water. No randomization of mice was used. The investigators were not blinded to the experimental groups. All mouse experiments followed the ARRIVE guidelines (http://www.nc3rs.org.uk/ARRIVEpdf). All experimental protocols were approved by the Committee on the Use of Live Animals in Teaching and Research at the University of Hong Kong (Ref. No. 5366-20).

### 4.3 Animal models

To establish the HFD-induced obesity model, 6-week-old male or female AT-Sdc4 KO and WT control mice were fed a standard chow diet (STC) or high-fat diet (HFD) with 45 kcal% of fat for 14 weeks, and the body weight was measured weekly. For the cold challenge study, 8-week-old male AT-Sdc4 KO and WT control mice were maintained in the temperature-controlled chamber for 7 days at 30 °C, followed by either 30 °C or 6 °C for another 2 or 7 days. Fat mass and lean mass were analyzed by the Minispec Body Composition Analyzer (Minispec LF Series, USA).

### 4.4 Indirect calorimetry analysis

Whole-body rate of oxygen consumption (VO_2_), carbon dioxide production (VCO_2_), energy expenditure, and locomotor activity were constantly assessed using Indirect Calorimetry with the Columbus Comprehensive Lab Animal Monitoring System (CLAMS, Columbus, USA) as previously described. Briefly, 6-week-old AT-Sdc4 KO mice and their littermates fed with STC or HFD for 14 weeks were acclimated to CLAMS cages individually with food and water *ad libitum* for 48 h. For calculation of energy expenditure: Energy Expenditure (Heat production) = calorific value (Cv) x VO_2_ = (3.815 + 1.232 × RER) × VO_2_ (Kcal h^−1^) [83]. Data was calculated by normalizing with lean mass. Food intake was calculated by measuring food consumption and the number of days.

### 4.5 Histological analysis

For H&E staining, adipose tissues were fixed in 4% PFA for 48 h at room temperature, embedded in paraffin, and sectioned to 5 μm. Deparaffinized and dehydrated sections were stained with hematoxylin and eosin to detect histopathological changes. Adipocyte sizes were analyzed by Image J software. Frozen liver sections were subjected to Oil Red O staining (ORO staining) to visualize lipid droplets. Briefly, the tissues were cut into 8 μm slices and then stained for lipids according to the standard ORO staining protocol. The area of positive staining for ORO was calculated as a percentage of the total section area by software Image J.

### 4.6 Glucose tolerance and insulin tolerance test

For glucose tolerance test, mice were housed in clean cages and fasting for 16 h before being intraperitoneally injected with d-glucose (1 g/kg). Blood glucose concentrations were then monitored at 0, 15, 30, 45, 60, 90, and 120 min after glucose injection. For the insulin tolerance test, mice were fasted for 6 h before intraperitoneal administration of human insulin (1 U/kg). Blood glucose was measured at 0, 30, 60, 90, and 120 min after insulin injection. Blood levels were measured using a glucometer and corresponding test strips (Sinocare, CN).

### 4.7 Seahorse analysis

Ex vivo oxygen consumption in adipose tissues was performed using a Seahorse XFe24 extracellular flux analyzer (Agilent Technologies) as previously described. Briefly, fresh sWAT or BAT collected from male AT-Sdc4 KO mice and their littermates fed with STC or HFD for 14 weeks was cut into 2 mm^3^ pieces before being placed in each well of a Seahorse XF24 Islet Capture plate. The minced tissues were equilibrated in DMEM without NaHCO_3_ (pH 7.4) for 1 h at 37 °C without CO_2_. The basal oxygen consumption rate (OCR) was measured and normalized with tissue weight.

### 4.8 Single-cell RNA-sequencing database analysis

We used the publicly available metadata (GSE176171) to characterize dietary-relevant transcriptional signatures (STC or HFD) in single-cell expression levels. For mouse adipocyte data in sWAT and eWAT, refer to the original paper [31]. Briefly, data were scaled by regressing out the nUMIs and percentage of mitochondrial gene expression in R package Seurat (v4.3.0). The dimensional reduction was conducted using the RunUMAP function. Cells were further identified as STC-sWAT (4980 cells), STC-eWAT (5483 cells), HFD-sWAT (9183 cells), and HFD-eWAT (2137 cells) based on the annotation from Emont et al. Subsequent expression analyses of Sdc4 (feature plots and violin plots) were performed using the Seurat function FeaturePlot and VlnPlot with default parameters.

### 4.9 RNA-sequencing analysis

High-throughput sequencing libraries were generated using 1 μg of total RNA extracted from eWAT samples obtained from AT-Sdc4 KO mice and WT controls fed with STC or HFD for 14 weeks. Each group consisted of four mice. To obtain sequencing data, the libraries with different indexes were multiplexed and loaded onto an Illumina HiSeq, Illumina Novaseq, or MGI2000 instrument. Prior to alignment, the reference genome sequences were indexed using Hisat2. Principal component analysis (PCA) was analyzed using the FactoMineR package (version 1.34). Differential expression analysis was conducted using the DESeq2 package. An adjusted p-value (p.adj) threshold of <0.05 was employed to identify differentially expressed genes (DEGs). The DEGs were visualized and clustered using the heatmap function from the heatmap package (version 1.0.12) in R. To gain insights into biological processes and perform gene set enrichment analysis (GSEA), Gene Ontology Biological Process (GOBP) enrichment analysis was conducted using the cluster Profiler package (version 4.6.0) in R. For GSEA, an Absolute Normalized Enrichment Score (NES) cutoff of ≥1 and a false discovery rate (FDR) q-value cutoff of <0.05 were employed to determine the significance of gene expression changes.

### 4.10 SCFAs measurement

Serums were collected from AT-Sdc4 KO mice and WT mice fed with HFD for 14 weeks. For sample preparation, 100 μl of the serum sample was mixed with 200 μl of 30 mM HCl, along with 50 μM of the internal standard, 3-methyl valeric acid. To extract the sample, 300 μL of Methyl tert-Butyl Ether (MTBE) was added to the sample mixture, followed by vortexing for 10 s to emulsify the components. Subsequently, phase separation was carried out, and 1 μL of the upper layer MTBE was collected for injection into the GC–MS/MS system. Data analysis was performed using the Agilent MassHunter Workstation Quantitative Analysis Software.

### 4.11 Rectal temperature measurement

The rectal temperatures of AT-Sdc4 KO mice and their littermates were measured by a thermometer with a rectal probe during cold exposure for 0, 1, 2, 4, 6, and 8 h (Model 4610 Precision Thermometer, Measurement Specialties, USA).

### 4.12 Enzyme-linked immunosorbent assay (ELISA)

Serum samples from STC- or HFD-fed AT-Sdc4 KO and WT controls were collected, and the concentrations of shed Sdc4 in serum were determined using the Mouse Syndecan-4 ELISA kit (Novus, USA) according to the manufacturer's instructions. The absorbance values were measured at 450 nm using a microplate reader.

### 4.13 Lipolysis assays

For ex vivo lipolysis assay, sWAT and eWAT isolated from male AT-Sdc4 KO mice and their littermates were dissected into ∼3 mm diameter pieces in cold PBS. WAT explants were incubated at 37 °C for 2 h in Krebs–Ringer buffer (30 mM HEPES, 10 mM NaHCO_3_, 4 mM K_3_PO_4_, 120 mM NaCl, 1 mM MgSO_4_, 1 mM CaCl_2_), and then stimulated with isoproterenol (10 nM) for 3 h. Media samples were collected at desired time point. To determine lipolysis in cultured cells, adipocytes were treated with RbSdc4 (100 ng/ml) in the presence or absence of 1 μM HSL inhibitor, HSL–IN–1, for 24 h. Afterward, 10 μM FSK was added to the serum-free culture medium containing 2 % fatty acid-free BSA for 4 h and FFAs released into the medium were examined. To determine FFA and glycerol release into serum during cold exposure, serum of AT-Sdc4 KO and control littermates was collected at 0, 1, 2, 4, and 6 h during cold stimulation for analysis. Following the instructions of the manufacturer, FFAs were quantified using the Free fatty acid assay kit (Abcam, USA), and glycerol was quantified using the Free glycerol assay kit (Abcam, USA).

### 4.14 Isolation, culture, and differentiation of adipocytes

Subcutaneous white adipose tissue (sWAT) obtained from male 6-week-old AT-Sdc4 KO and WT control mice was dissected into small fragments of approximately 0.5 mm^3^ in size and digested with 1.5 mg/ml collagenase I at 37 °C incubator for 45 min. The digestion mixture was pipetted up and down and then passed through a 70 μm strainer to remove any tissue debris. After centrifugation at 800×*g* for 10 min at 4 °C, the cell pellet used as SVF was carefully washed and cultured in Dulbecco's Modified Eagle Medium (DMEM) with 10% fetal bovine serum (FBS) and 1% penicillin and streptomycin. To differentiate SVFs into mature adipocytes, the differentiation cocktail including 10 μg/ml insulin, 0.5 mM 3-isobutyl-1-methylxanthine, 1 μM dexamethasone, and 1 μM rosiglitazone was added to the SVFs for the first 2 days, followed by 10 μg/ml insulin treatment for another 6 days.

### 4.15 Cell treatment

SVFs-derived adipocytes were treated with Sdc4 recombinant protein (RbSdc4) and/or FGF2 at different doses for 24 h. To inhibit FGFR1 activation, adipocytes were treated with FGFR1-specific inhibitor (PD-166866, 0.5 or 1 μM) for 24 h.

### 4.16 RT-qPCR analysis

Total RNA was isolated from cells and tissues using Trizol reagent according to the manufacturer's instructions. The purity and concentration of the total RNA were determined by a spectrophotometer at 260 and 280 nm. The absorption ratios of all samples were between 1.8 and 2.0. 1 μg total RNA was converted to cDNA using PrimeScript RT reagent kit (Takara). Quantitative real-time PCR (RT-qPCR) was performed on a StepOne System (Applied Biosystems) using TB Green® PrimeScript™ RT-PCR Kit II (Takara). The house-keeping gene (*36b4*) was used as a reference. Fold changes in expression levels were calculated according to the value of 2^-ΔΔCt^. The primer sequences are listed in Supplementary Table 1.

### 4.17 Protein extraction and western blot analysis

Proteins were extracted from adipocytes or adipose tissues using freshly prepared RIPA buffer containing the protease inhibitor cocktail (Proteinase inhibitor, Na_3_VO_4_ and NaF). Protein concentrations were determined using Pierce BCA Protein Assay Reagent (Thermo Fisher). Total proteins were separated by SDS-PAGE and transferred onto polyvinylidene fluoride (PVDF) membranes (Millipore Corporation). After blocking with 10% fat-free milk for 1 h at room temperature, the membranes were probed with primary antibodies at 4 °C overnight, followed by incubation with corresponding horseradish peroxidase (HRP)-conjugated secondary antibodies for 1 h at room temperature. All antibodies used in the study are shown in Supplementary Table 2. The protein bands were visualized with enhanced chemiluminescence reagent (Bio-Rad) and scanned with ChemiDoc™ MP Imaging System (Bio-Rad). The results shown in the figures are representative of results from at least three independent experiments. Protein band densities were quantified using Image J software.

### 4.18 Co-immunoprecipitation

Total proteins were extracted from eWAT of male AT-Sdc4 KO mice and WT mice fed with STC or HFD for 14 weeks. 500 μg total protein was incubated with anti-rabbit Sdc4 antigen affinity-purified polyclonal antibody (5 μg/ml, Novus, USA) or anti-rabbit IgG (5 μg/ml, CST, USA) at 4 °C overnight, followed by precipitation with protein A/G Plus-Agarose beads (Santa Cruz, USA) at room temperature for 3 h with mixing. After the beads were washed five times with ice-cold lysis buffer, the immunoprecipitated complexes were collected for Western blot analysis.

### 4.19 Statistical analysis

All data were statistically analyzed in GraphPad Prism 9 (GraphPad Software Inc, USA). The quantitative analysis of H&E, Oil Red O, and western blot results were performed using Image J software, and each dot in the quantitative graphs represents a parallel independent biological sample test. All replicated experiments were biologically repeated at least three times. Data were presented as the mean ± standard error of the mean (SEM). Statistical significance was assessed by the Mann–Whitney *U* test, unpaired two-tailed Student's t-test, and one-way or two-way ANOVA followed by Bonferroni's multiple comparisons test. *P* value of <0.05 was considered statistically significant.

## CRediT authorship contribution statement

**Jiuyu Zong:** Writing – original draft, Project administration, Investigation. **Xiaoping Wu:** Investigation, Funding acquisition, Writing – review & editing. **Xiaowen Huang:** Investigation. **Lufengzi Yuan:** Investigation. **Kai Yuan:** Investigation. **Zixuan Zhang:** Investigation. **Mengxue Jiang:** Investigation. **Zhihui Ping:** Investigation. **Lai Yee Cheong:** Investigation. **Aimin Xu:** Supervision. **Ruby Lai Chong Hoo:** Project administration, Supervision, Funding acquisition, Writing – review & editing.

## Declaration

Generative AI is not used in writing.

## Declaration of competing interest

All the authors have declared that no conflict of interest exists.

## Data Availability

Data will be made available on request.
