# Supplementary material for: Adipocyte-derived shed Syndecan-4 suppresses lipolysis contributing to impaired adipose tissue browning and adaptive thermogenesis
Source: Mol Metab. 2025 Apr 1;96:102133. doi: 10.1016/j.molmet.2025.102133 (PMC12004711; doi:10.1016/j.molmet.2025.102133)
Supplement: Multimedia component 1 [file mmc1.docx]

**Table S1: Sequence of primers for qPCR and genotyping**

| **Name** | **Sequence (5’ to 3’)** | |
| --- | --- | --- |
| *Sdc4* | Forward | AGCCCTTGGTGCCACTGGATA |
|  | Reverse | GGCAGTGCTGGACATGGATAC |
| *Hsl* | Forward | TGGCACACCATTTTGACCTG |
|  | Reverse | TTGCGGTTAGAAGCCACATAG |
| *Atgl* | Forward | AACACCAGCATCCAGTTCAA |
|  | Reverse | GGTTCAGTAGGCCATTCCTC |
| *Cpt1α* | Forward | GGCAGAAGAGATGGCGGTCGATG |
|  | Reverse | CCCCAAGTCAACGGCAGAGCAGA |
| *Lcad* | Forward | GCATCAACATCGCAGAGAAA |
|  | Reverse | GGCTATGGCACCGATACACT |
| *Mcad* | Forward | GCATCAACATCGCAGAGAAA |
|  | Reverse | CATTGTCCAAAAGCCAAACC |
| *Pparα* | Forward | TTTCGGCGAACTATTCGGCTG |
|  | Reverse | GGCATTTGTTCCGGTTCTTCTT |
| *Tnfα* | Forward | ACGGCATGGATCTCAAAGAC |
|  | Reverse | AGATAGCAAATCGGCTGACG |
| *Il6* | Forward | AGTTGCCTTCTTGGGACTGA |
|  | Reverse | CAGAATTGCCATTGCACAAC |
| *Mcp1* | Forward | TTAAAAACCTGGATCGGAACCAA |
|  | Reverse | GCATTAGCTTCAGATTTACGGGT |
| *Il1β* | Forward | GCCCATCCTCTGTGACTCAT |
|  | Reverse | AGGCCACAGGTATTTTGTCG |
| *Ucp1* | Forward | ACTGCCACACCTCCAGTCATT |
|  | Reverse | CTTTGCCTCACTCAGGATTGG |
| *Dio2* | Forward | CAGTGTGGTGCACGTCTCCAATC |
|  | Reverse | TGAACCAAAGTTGACCACCAG |
| *Cidea* | Forward | TGCTCTTCTGT A TCGCCCAGT |
|  | Reverse | GCCGTGTTAAGGAATCTGCTG |
| *Cox8a* | Forward | GTTCCAGCAGGATGGGTCTTAG |
|  | Reverse | TTCATGCTGCGGAGCTCTT |
| *Adamts1* | Forward | CTGGCAGAAACAACACAACAG |
|  | Reverse | TGAATTGGGCCATGTGTTTAAC |
| *Adamts4* | Forward | GCATTCCATGGTACAGGGTTA |
|  | Reverse | AGTTGACAGGGTTTCGGATG |
| *Mmp9* | Forward | TCTGTATGGTCGTGGCTCTAA |
|  | Reverse | GGAGGTATAGTGGGACACATAGT |
| *Prdm16* | Forward | CAGCACGGTGAAGCCATTC |
|  | Reverse | GCGTGCATCCGCTTGTG |
| *Pgc1α* | Forward | AGCCGTGACCACTGACAACGAG |
|  | Reverse | GCTGCATGGTTCTGAGTGCTAAG |
| *Pparγ* | Forward | GCCTGCGGAAGCCCTTTGGTGAC |
|  | Reverse | TTGGCGAACAGCTGGGAGGACTC |
| *Adiponectin* | Forward | GGAGAGAAAGGAGATGCAGGT |
|  | Reverse | CTTTCCTGCCAGGGGTTC |
| *36b4* | Forward | GCTCCAAGCAGATGCAGCA |
|  | Reverse | CCGGATGTGAGGCAGCAG |
| Sdc4 flox  (Neo-del) | Forward | GCATCCCTTATCCGGAGTTGAT |
|  | Reverse | AACTAGGGGACACACTAACCCTA |
| Adipoq-Cre | Forward | GGATGTGCCATGTGAGTCTG |
|  | Reverse | ACGGACAGAAGCATTTTCCA |

**Table S2: Key resources table**

| **Chemicals, peptides, and recombinant proteins** | | |
| --- | --- | --- |
| Protein A/G PLUS-Agarose | Santa Cruz | Cat: sc2003 |
| Restore^TM^ Western Blot Stripping Buffer | Thermo Scientific | Cat: 21059 |
| PageRuler^TM^ Prestained Protein Ladder | Thermo Scientific | Cat: 26617 |
| Isopropanol | Thermo Scientific | Cat: AR1162 |
| Insulin | Roche | 11061-680 |
| Clarity^TM^ Western ECL Substrate | Bio-Rad | Cat: 170-5060 |
| Protease inhibitor cocktail | Biomake | Cat: B14001 |
| Fetal Bovine Serum (FBS) | Gibco | Cat: 10270106 |
| Collagenase I | Gibco | Cat: 17100017 |
| Dulbecco’s Modified Eagle Medium (DMEM) | Gibco | Cat: 12100046 |
| TRIzol | Takara | Cat: 108952 |
| Chloroform | RciLabscan | Cat: AR1027E |
| Oil Red O | Sigma-Aldrich | Cat: 1320-06-5 |
| Isoproterenol | Sigma-Aldrich | Cat: I6504 |
| Forskolin (FSK) | Sigma-Aldrich | Cat: F3917 |
| Free fatty acid BSA | Sigma-Aldrich | Cat: A8806 |
| Rosiglitazone | Sigma-Aldrich | Cat: 122320-734 |
| Dexamethasone | Sigma-Aldrich | Cat: 50-02-2 |
| 3-Isobutyl-1-methylxanthine | Sigma-Aldrich | Cat: 28822-584 |
| Hematoxylin | Sigma-Aldrich | Cat: MFCD00078111 |
| Eosin | Sigma-Aldrich | Cat: HT110332-1L |
| Recombinant Mouse Syndecan-4 protein | R&D Systems | Cat: CF6267-SD-050 |
| Recombinant Mouse FGF2 protein | R&D Systems | Cat: 3139-FB |
| FGFR1 inhibitor (PD-166866) | MedChemExpress | Cat: HY-101296 |
| HSL-IN-1 | MedChemExpress | HY-101509 |
| Pierce^TM^ BCA Protein Assay Kits | Thermo Scientific | Cat: 23225 |
| PrimeScript^TM^ RT reagent Kit | Takara | Cat: RR037A |
| TB Green Premix Ex Taq II | Takara | Cat: RR82LR |
| Mouse Syndecan-4 ELISA Kit | Novus | Cat: NBP2-76612 |
| Triglyceride Liquicolor | Stanbio | Cat: 2100-430 |
| Cholesterol Liquicolor | Stanbio | Cat: 1010-430 |
| Free fatty acid assay kit | Abcam | Cat: ab65341 |
| Free glycerol assay kit | Abcam | Cat: ab5337 |
| PVDF transfer membrane | Merck | ISEQ00010 |
| Medical X-ray film | Fujifilm | Super RX-N |
| High Fat Diet (HFD; 45% kcal fat) | Research Diets | Cat: D12451 |
| 70 µm cell strainer | Corning | Cat: 431751 |
| Glucometer and strips | Sinocare | Cat: 100012143343 |
| V28 TC-Treated Cell Culture Microplates | Agilent | Cat: 100882-004 |
| Islet Capture Microplates | Agilent | Cat: 101122-100 |
| **Antibodies** |  |  |
| Rabbit anti-Syndecan-4 | Novus | Cat: NB110-4155 |
| Rabbit anti-UCP1 | Abcam | Cat: ab23841 |
| Mouse anti-FGF2 | Santa Cruz | Cat: sc-365106 |
| Rabbit anti-phospho-HSL (Ser660) | CST | Cat: 45804 |
| Rabbit anti-HSL | CST | Cat: 4107 |
| Rabbit anti-HSP90 | CST | Cat: 4875 |
| Rabbit anti-ATGL | CST | Cat: 2138 |
| Phospho-PDE4D PKA site (Ser54) | Fabgennix | PPD4-440AP |
| PDE4D Polyclonal antibody | Proteintech | 12918-1-AP |
| Anti-rabbit IgG HRP-linked | CST | Cat: 7074 |
| Anti-mouse IgG HRP-linked | CST | Cat: 7076 |
| Rabbit Control IgG | CST | Cat: 2729 |
| **Experimental models: Organisms/strains** | | |
| Mouse: wild type C57BL/6N | Centre for Comparative Medicine Research | The University of Hong Kong |
| Mouse: Sdc4^f/f^ | Cyagen | TOBS-20071-01 |
| Mouse: Adiponectin-Cre | The Jackson Laboratory | 010803 |
| **Deposited data** |  |  |
| Original RNA-seq data | This paper | N/A |
| Single-cell atlas of mouse white adipose tissue | Online database | GSE176171 |
| **Software and algorithms** |  |  |
| Image J | National institutes of Health | https://imagej.net/ij/ |
| Graphpad 9.0 | Graphpad | https://www.graphpad.com/ |
| Hisat2 | Johns Hopkins University | https://daehwankimlab.github.io/hisat2/ |
| R Studio | R Foundation | https://www.r-project.org/ |
