# Supplementary material for: Adipocyte-derived shed Syndecan-4 suppresses lipolysis contributing to impaired adipose tissue browning and adaptive thermogenesis
Source: Mol Metab. 2025 Apr 1;96:102133. doi: 10.1016/j.molmet.2025.102133 (PMC12004711; doi:10.1016/j.molmet.2025.102133)
Supplement: Multimedia component 2 [file mmc2.docx]

**Supplementary Figure 1**


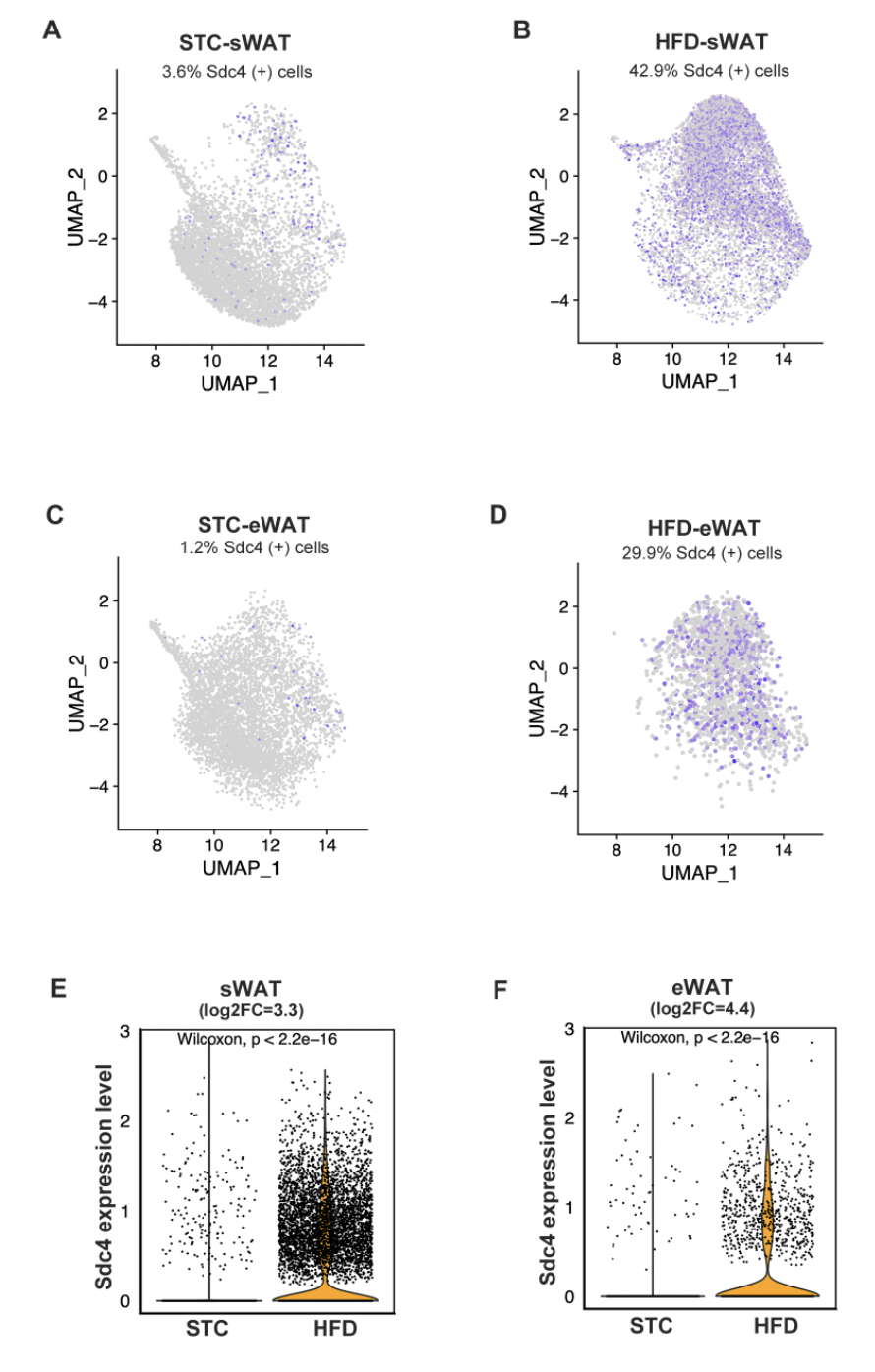


**Supplementary Figure 1. The population of Sdc4-expressing adipocytes is increased in DIO mice.**

Analysis of single RNA sequencing dataset (GSE176171) of sWAT and eWAT of 19-week-old HFD or STC fed male C57Bl/6J mice. **(A-D)** The UMAP visualization of the single cells with feature plots depicting Sdc4 expression within the adipocyte population in (A-B) sWAT and (C-D) eWAT. **(E-F)** Violin plots indicating the mRNA expression level of Sdc4 in the adipocytes of (E) sWAT and (F) eWAT from the single cell level. Statistical significance was analyzed by the Wilcoxon test (E and F).

**Supplementary Figure 2**

**
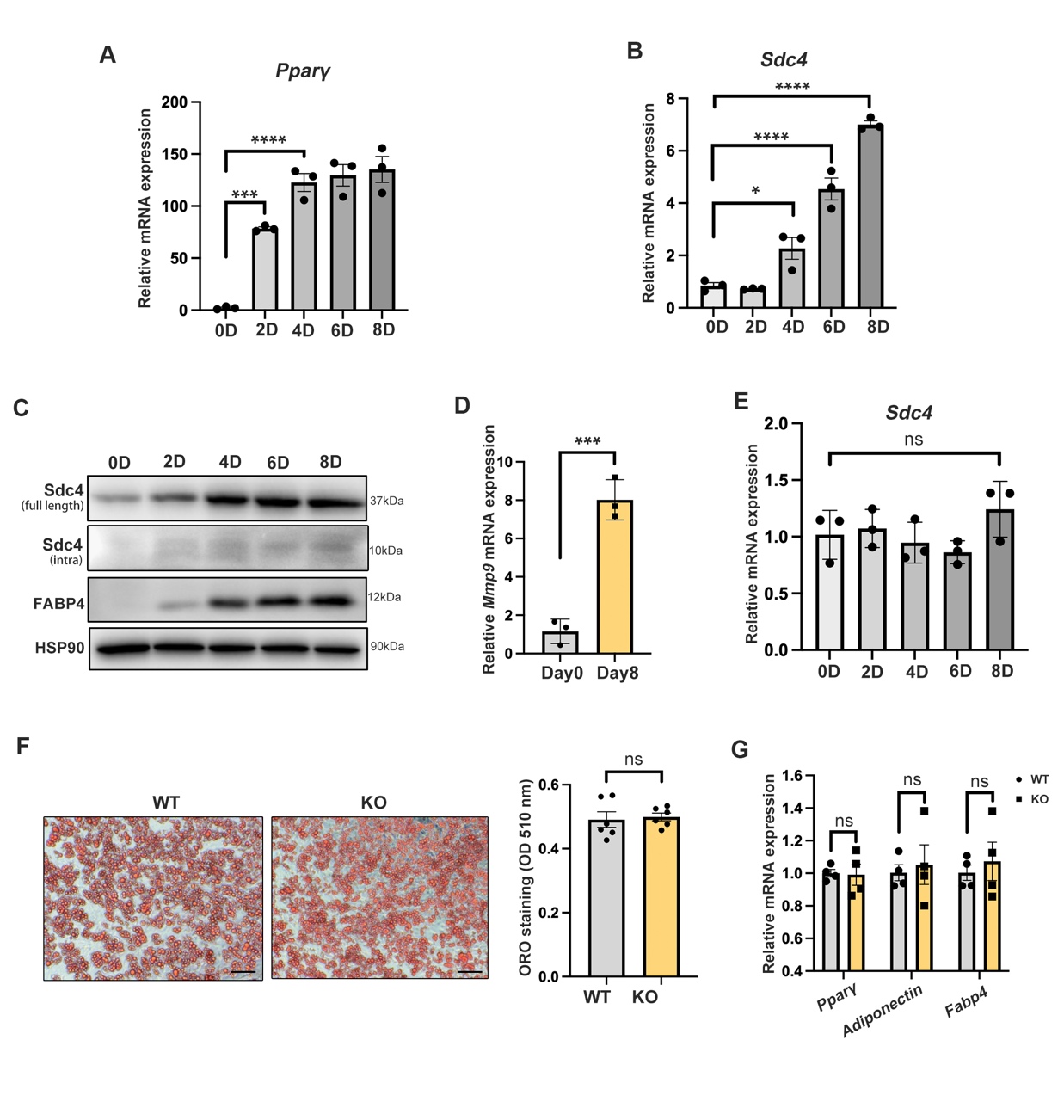
**

**Supplementary Figure 2. The expression of Sdc4 is increased along white adipocyte differentiation.**

Sdc4 KO and WT white adipocytes were differentiated from SVFs isolated from AT-Sdc4 KO and WT mice. **(A-B)** The relative mRNA abundance of adipogenic marker (*Pparγ*) and *Sdc4* during 0 to 8 days differentiation of WT white adipocytes (n = 3). **(C)** Representative immunoblots of protein abundance of full-length intact Sdc4, intra Sdc4 (the remaining part after shedding), adipogenic marker FABP4, and HSP90 during differentiation of WT white adipocytes. **(D)** The relative mRNA abundance of *Mmp9* at 0 and 8 days of white adipocyte differentiation (n = 3). **(E)** The relative mRNA abundance of *Sdc4* during 0 to 8 days differentiation of Sdc4 KO adipocytes (n = 3). **(F)** The representative images of Oil Red O staining of WT or Sdc4 KO white adipocytes after 8 days of differentiation. The right panel is the quantification of neutral lipid contents (n = 6, scale bar = 100μm). **(G)** The relative mRNA abundance of adipogenic markers (*Pparγ*, *Adiponectin*, and *Fabp4*) in 8-day-differentiated WT and Sdc4 KO white adipocytes (n = 4). Data is presented as mean ± SEM. Statistical significance was analyzed by the one-way ANOVA (A, B, and E), the Mann-Whitney *U* test (D), or the unpaired two-tailed Student’s *t* test (F and G).

**Supplementary Figure 3**

**
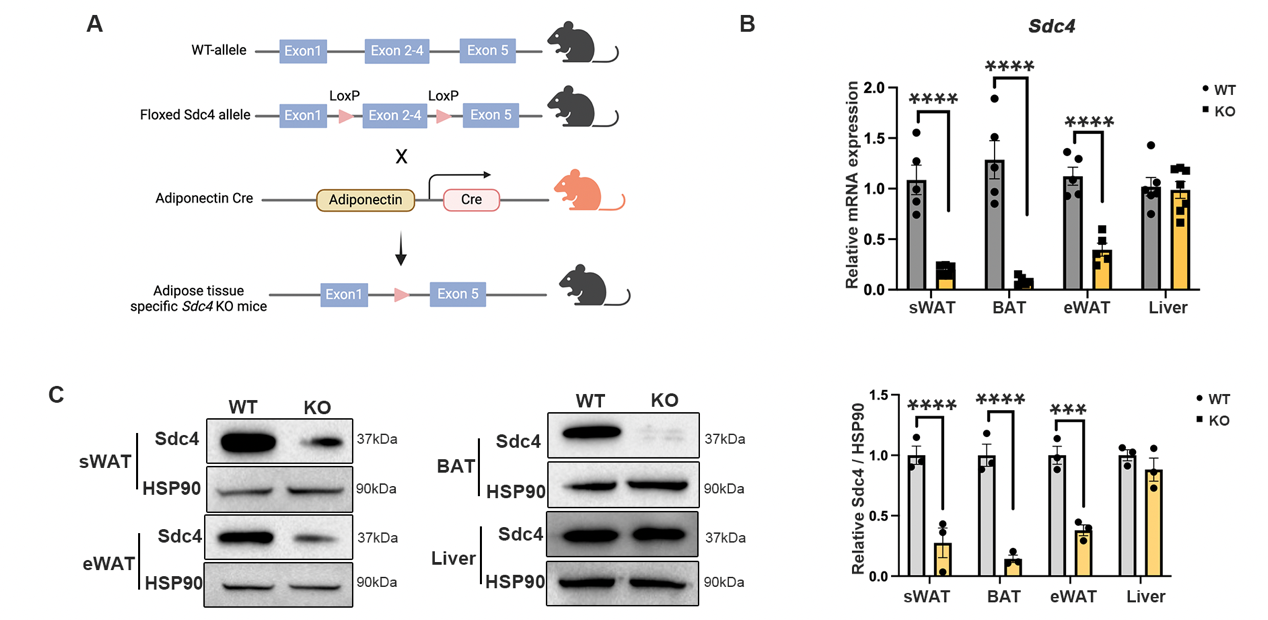
**

**Supplementary Figure 3. Generation and identification of adipocyte-specific Sdc4 knockout mice. (A)** Schematic diagram showing the generation of Sdc4 flox/flox mice (WT mice) and adipocyte-specific Sdc4 knockout (AT-Sdc4 KO) mice. **(B)** The relative mRNA abundance of *Sdc4* in different tissues from AT-Sdc4 KO mice and WT mice (n=5). **(C)** Representative immunoblots of protein abundance of full-length Sdc4 and HSP90 in sWAT, eWAT, BAT, and liver of AT-Sdc4 KO mice and WT mice. The right panel is the quantification of Sdc4 band intensity normalized to HSP90 (n = 3). Data is presented as mean ± SEM. Statistical significance was analyzed by the unpaired two-tailed Student’s *t* test (B) or the Mann-Whitney *U* test (C). ****p*<0.001, *****p*<0.0001.

**Supplementary Figure 4**

**
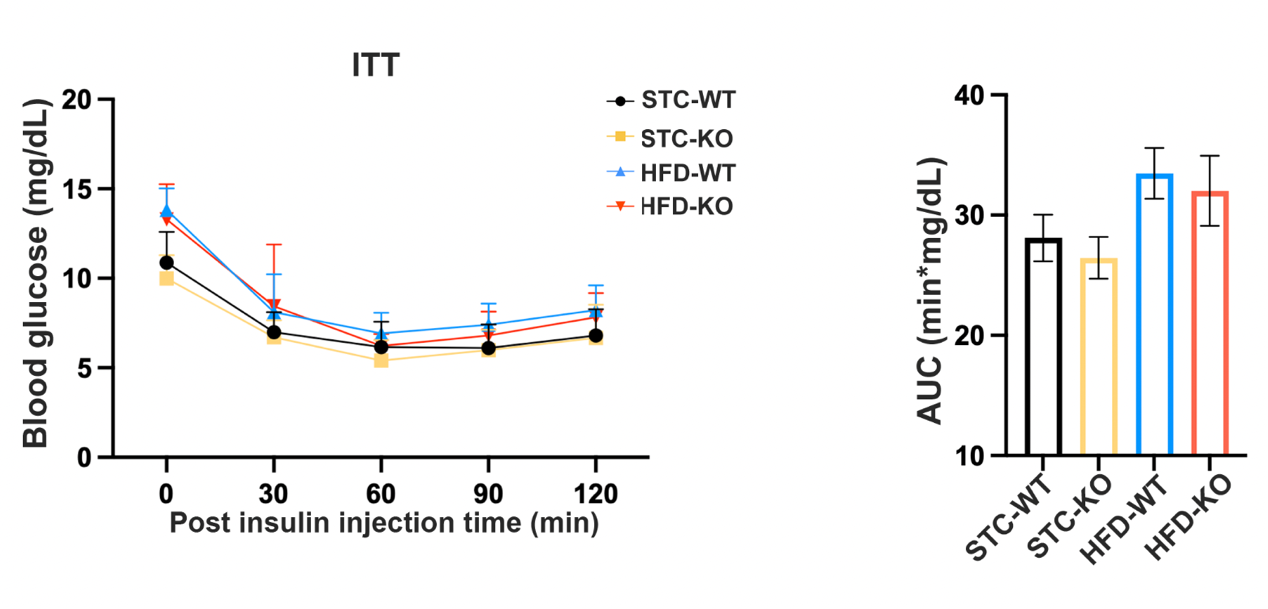
**

**Supplementary Figure 4. The effects of adipocyte-Sdc4 deficiency on insulin sensitivity of DIO mice.** Six-week-old male AT-Sdc4 KO mice and WT mice were subjected to HFD or STC feeding for 14 weeks. Insulin tolerance test (left), and AUC was calculated (right) (n = 6). Data are expressed as the mean ± SEM. Statistical significance was assessed by two-way ANOVA.

**Supplementary Figure 5**

**
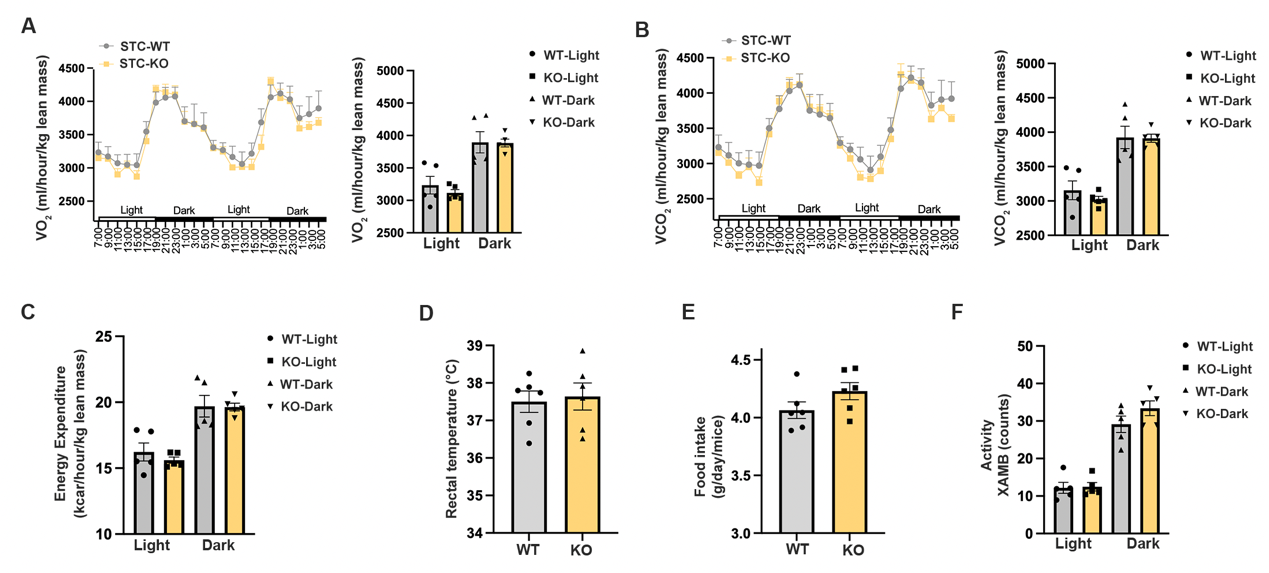
**

**Supplementary Figure 5. Adipocyte-specific Sdc4 deficiency does not affect energy expenditure in STC-fed mice**

Six-week-old male AT-Sdc4 KO mice and their relative WT littermates were subjected to STC feeding for 14 weeks, followed by metabolic cage assessment at 48 hours. **(A-B)** The light (day) and dark (night) rate of (A) oxygen consumption (VO_2_) and (B) carbon dioxide (VCO_2_) production in mice. The right panels are the quantification of VO_2_ consumption and VCO_2_ production shown in (A) and (B), respectively (n = 5). **(C)** The calculated energy expenditure in mice (n = 5). **(D)** The rectal temperature of mice measured at the 48 h endpoint (n = 6). **(E-F)** The (E) food intake and (F) locomotive activity in mice (n = 5). Data is presented as mean ± SEM. Statistical significance was assessed by two-way ANOVA (A, B, C, and F) or unpaired two-tailed Student’s *t* test (D-E).

**Supplementary Figure 6**

**
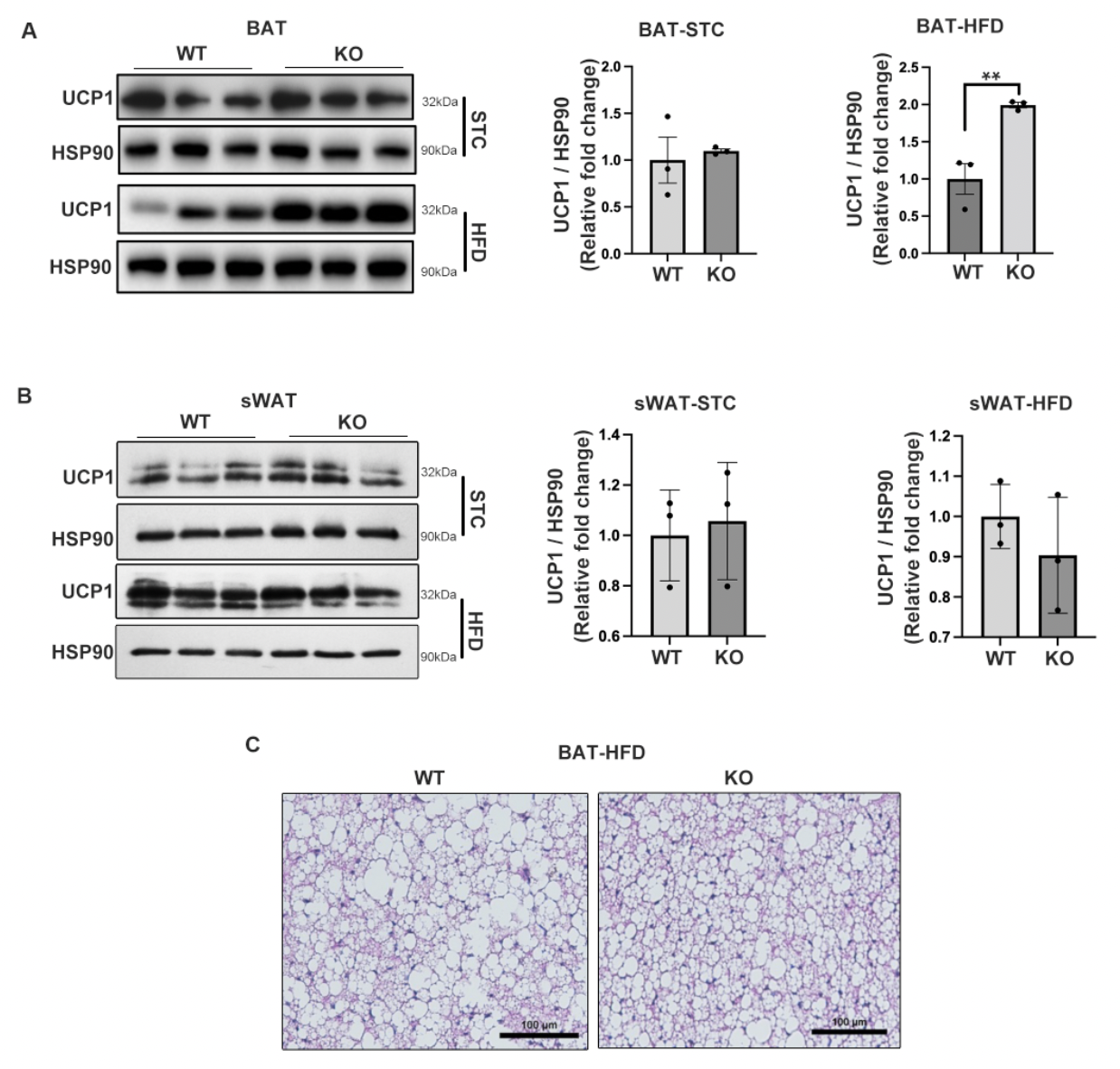
**

**Supplementary Figure 6. Adipocyte-specific Sdc4 knockout promotes UCP1 expression in BAT but not sWAT of DIO mice.**

Six-week-old male AT-Sdc4 KO mice and WT littermates were subjected to HFD or STC feeding for 14 weeks. **(A-B)** Representative immunoblots of protein abundance of UCP1 and HSP90 in (A) BAT and (B) sWAT of mice. The right panels are the quantification of UCP1 band intensity normalized to HSP90 (n = 3). **(C)** The representative images of H&E staining of BAT of HFD-fed WT and AT-Sdc4 KO mice (scale bar = 100 µm). Data is presented as mean ± SEM. Statistical significance was assessed by the Mann-Whitney *U* test (A and B).

**Supplementary Figure 7**

**
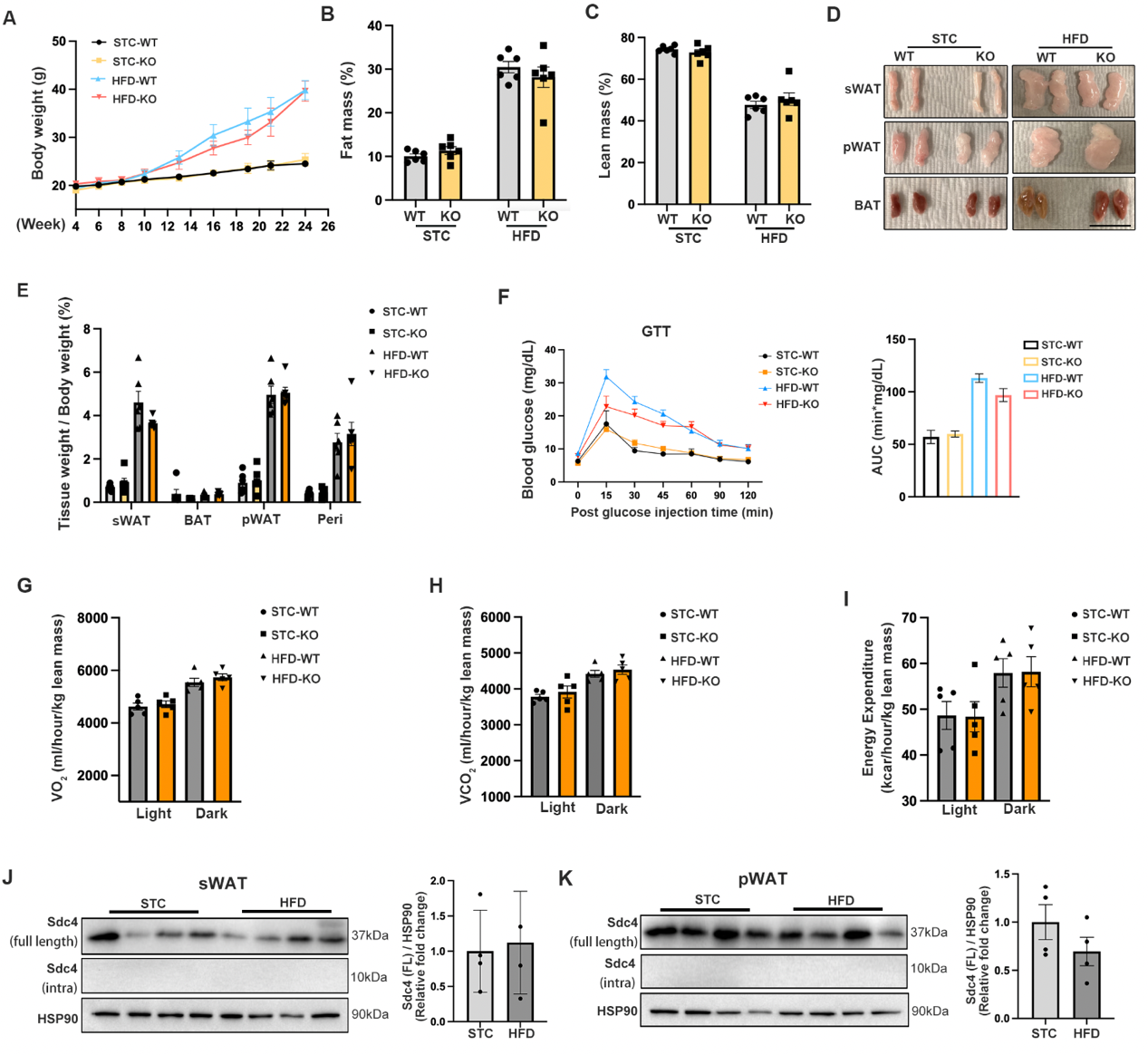
**

**Supplementary Figure 7. Adipocyte-specific Sdc4 deficiency does not cause metabolic difference in female mice subjected to diet-induced obesity.**

Six-week-old female AT-Sdc4 KO and WT control mice were subjected to HFD or STC feeding for 24 weeks. **(A)** Dynamic change of mouse body weight (n = 6). **(B-C)** The (B) fat mass percentage and (C) lean mass percentage of mice (n = 6). **(D-E)** The (D) representative images of mouse sWAT, pWAT, and BAT (scale bar = 1 cm) and (E) relative weights of various fat depots (n = 6). **(F)** Glucose tolerance test (left) and calculated areas under the curve (AUC, right) (n = 6). **(G-I)** The light (day) and dark (night) rate of oxygen consumption (VO_2_) and (H) carbon dioxide production (VCO_2_), and (I) energy expenditure of HFD-fed female AT-Sdc4 KO and WT mice assessed over 48 h using metabolic cage (n=5). **(J-K)** Representative immunoblots of protein abundance of full-length intact Sdc4, intra Sdc4 (the remaining part after shedding), and HSP90 in mouse (J) sWAT and (K) pWAT. The right panel is the quantification of Sdc4 band intensity normalized to HSP90 (n = 4). Data is presented as mean ± SEM. Statistical significance was assessed by the two-way ANOVA (A to I) or the Mann-Whitney *U* test (J and K). pWAT, perigonadal white adipose tissue; Peri, perirenal fat.

**Supplementary Figure 8**

**
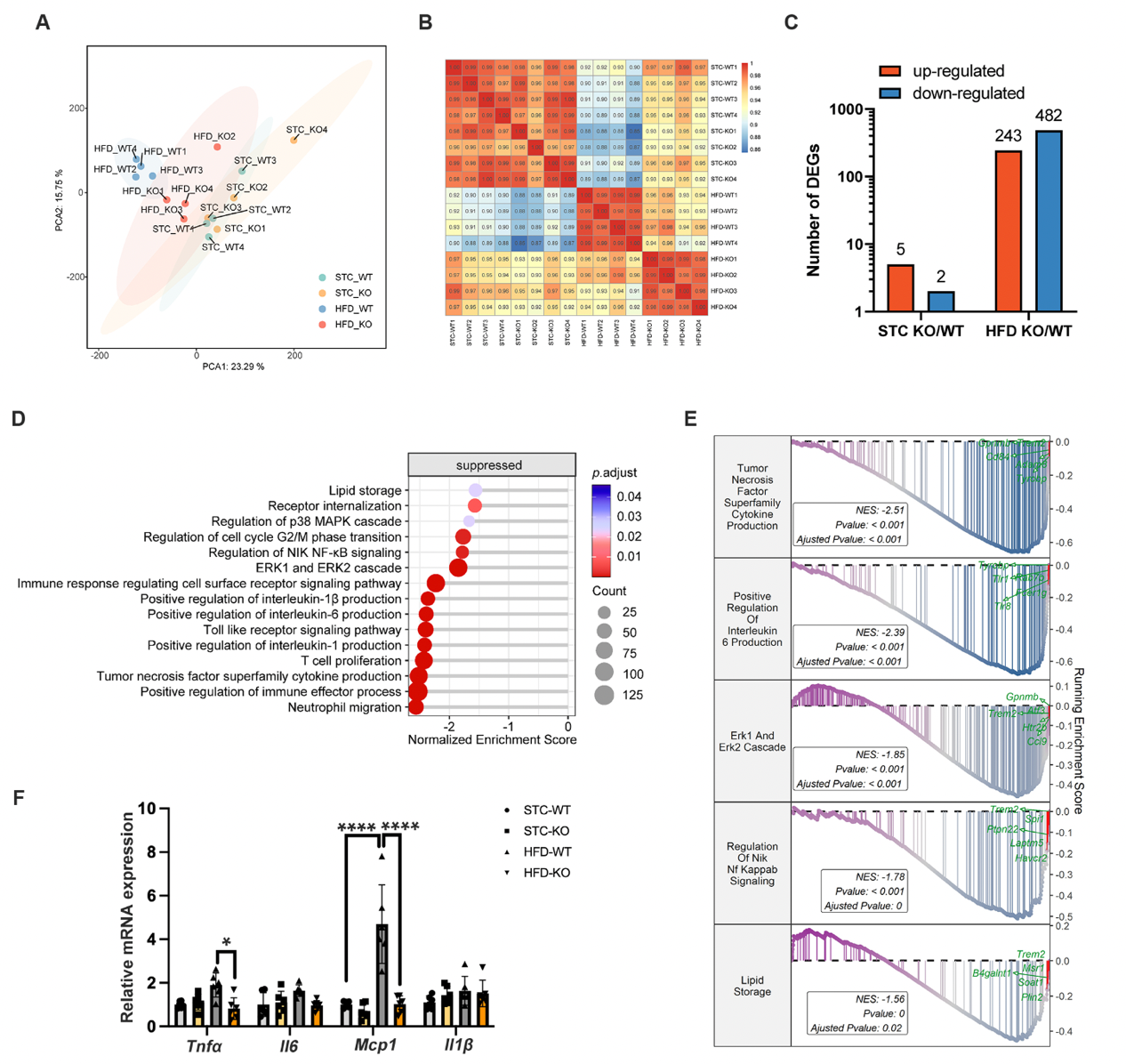
**

**Supplementary Figure 8. Sdc4 deficiency in adipocytes alleviates HFD-induced inflammation.** Six-week-old male AT-Sdc4 KO and WT mice were subjected to STC and HFD feeding for 14 weeks. The RNA of eWAT were extracted for RNA-seq analysis (n=4). **(A)** Principal-component analysis (PCA) of gene expression profiles of the STC-fed WT mice (STC-WT), STC-fed AT-Sdc4 KO mice (STC-KO), HFD-fed WT mice (HFD-WT), and HFD-fed AT-Sdc4 KO mice (HFD-KO). The ellipses indicate the 95% confidence band. **(B)** The gene expression correlation heatmap. **(C)** The number of differentially expressed genes (DEGs) from STC-KO mice compared to STC-WT mice and HFD-KO mice compared to HFD-WT mice, respectively. The upregulated and downregulated DEGs were estimated by DESeq2 (fold change >2, p.adj <0.05). **(D)** Gene set enrichment analysis (GSEA) of ontology-biological process pathways preferentially suppressed in the eWAT of HFD-fed AT-Sdc4 KO mice compared to WT mice (n=4). Terms were ranked based on the normalized enrichment score (NES). **(E)** The representative GSEA-scoring plots of activated GO-BP pathways in (D). The reported NES values and adjusted p values (p. adjust) were calculated with 1,000 permutations in the GSEA software. (**F**) The relative mRNA abundance of inflammatory genes in mouse eWAT. Data is presented as mean ± SEM. Statistical significance was analyzed by two-way ANOVA (F). **p*<0.05, *****p*<0.0001.

**Supplementary Figure 9**

**
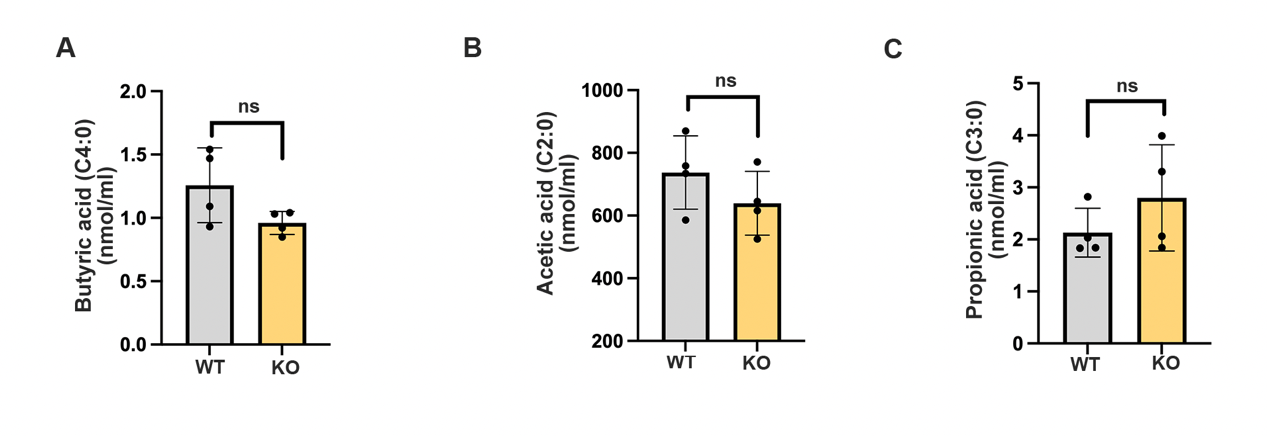
**

**Supplementary Figure 9. The concentrations of SCFAs in the serum of AT-Sdc4 KO mice and WT mice.**

Male AT-Sdc4 KO mice and WT mice were subjected to HFD feeding for 14 weeks. **(A-C)** The concentrations of SCFAs (butyrate, acetate, and propionate) in serum analyzed by GC-MS (n=4). Data is presented as mean ± SEM. Statistical significance was analyzed by the Mann-Whitney *U* test (A to C). ns, not significant.

**Supplementary Figure 10**

**
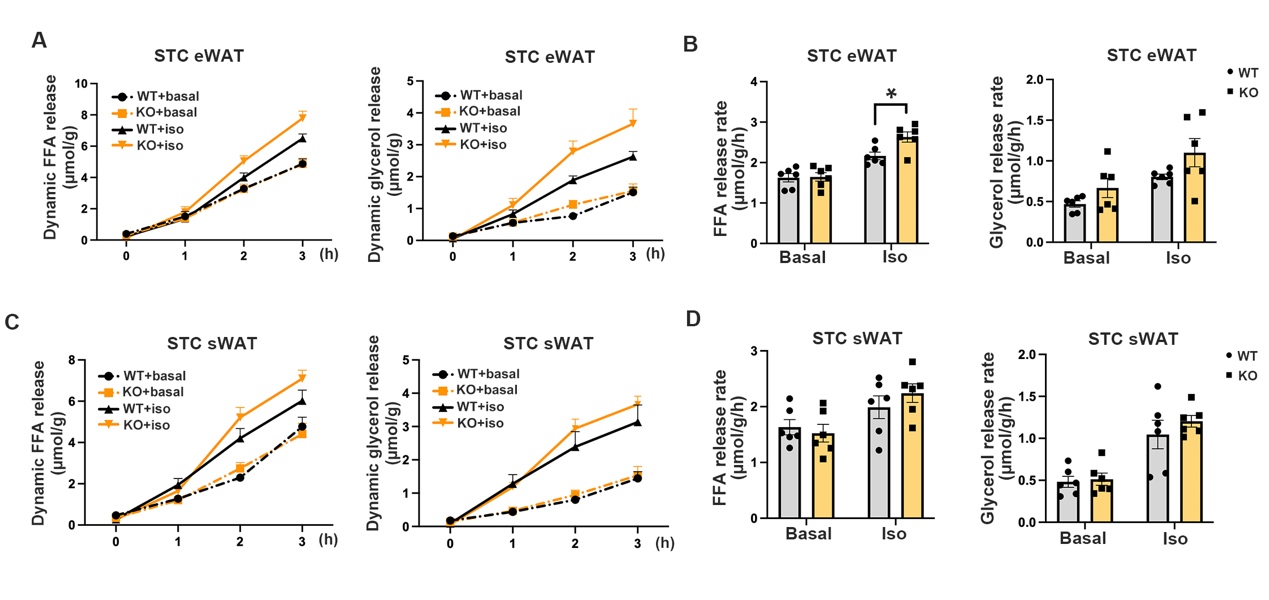
**

**Supplementary Figure 10. Adipocyte-specific Sdc4 deficiency mildly affects lipolysis under STC feeding conditions.**

Six-week-old male AT-Sdc4 KO mice and WT mice fed with 14 weeks of STC were injected with isoproterenol (Iso, 10 mg/kg body weight). **(A-B)** The (A) dynamic and (B) calculated FFA and glycerol releasing profile from eWAT (n = 6). **(C-D)** The (C) dynamic and (D) calculated FFA and glycerol releasing profile from sWAT (n = 6). Data is presented as mean ± SEM. Statistical significance was analyzed by the two-way ANOVA (B and D). **p*<0.05.

**Supplementary Figure 11**

**
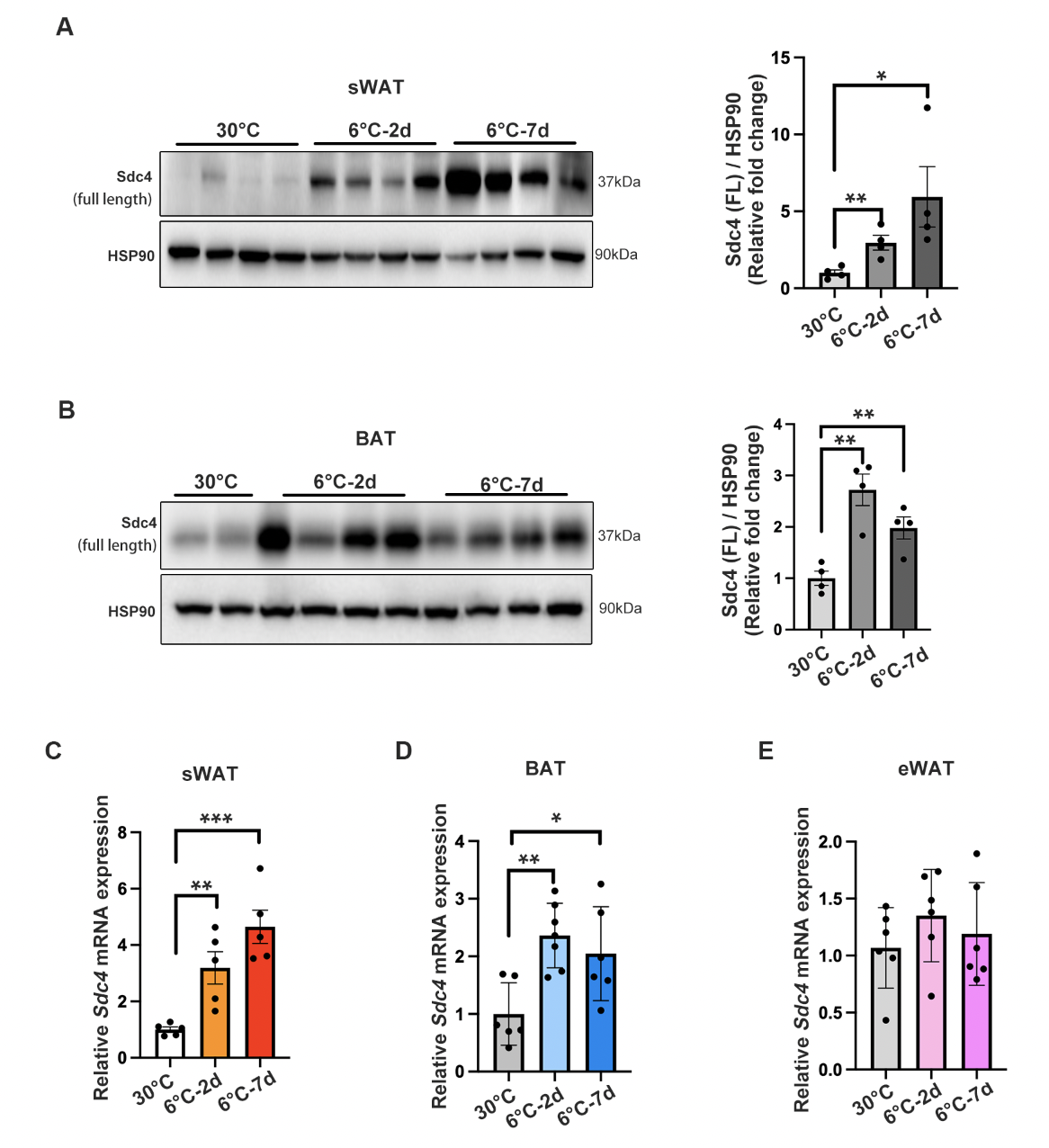
**

**Supplementary Figure 11. Sdc4 expression is increased in sWAT and BAT of mice subjected to cold challenge.**

Eight-week-old male C57BL/6N mice were subjected to 30 °C acclimation for 7 days, followed by 6 °C cold challenge for 2 or 7 days. **(A-B)** Representative immunoblots of the protein abundance of full-length Sdc4 and HSP90 in mouse (A) sWAT and (B) BAT. The right panels are the quantification of Sdc4 band intensity normalized to HSP90 (n = 4). **(C-E)** The relative mRNA abundance of *Sdc4* in mouse (C) sWAT, (D) BAT, and (E) eWAT (n = 6). Data is presented as mean ± SEM. Statistical significance was analyzed by the one-way ANOVA (A-E). **p*<0.05, ***p*<0.01.****p*<0.001.
